# Selective reduction and homologation of carbon monoxide by organometallic iron complexes

**DOI:** 10.1038/s41467-018-06242-w

**Published:** 2018-09-14

**Authors:** Helen R. Sharpe, Ana M. Geer, Laurence J. Taylor, Benjamin M. Gridley, Toby J. Blundell, Alexander J. Blake, E. Stephen Davies, William Lewis, Jonathan McMaster, David Robinson, Deborah L. Kays

**Affiliations:** 1 School of Chemistry, University of Nottingham, University Park, Nottingham, NG7 2RD UK; 2 Department of Chemistry and Forensics, School of Science and Technology, Nottingham Trent University, Nottingham, NG11 8NS UK; 3 Present Address: School of Chemistry, The University of Manchester, Oxford Road, Manchester, M13 9PL UK; 4 Present Address: Department of Geography, Strand Building, King’s College London, Strand Campus, London, WC2R 2LS UK

## Abstract

Carbon monoxide is a key C_1_ feedstock for the industrial production of hydrocarbons, where it is used to make millions of tonnes of chemicals, fuels, and solvents per annum. Many transition metal complexes can coordinate CO, but the formation of new C−C bonds in well-defined compounds from the scission and subsequent coupling of two or more CO moieties at a transition metal centre remains a challenge. Herein, we report the use of low-coordinate iron(II) complexes for the selective scission and homologation of CO affording unusual squaraines and iron carboxylates at ambient temperature and pressure. A modification of the ligand framework allows for the isolation and structural characterisation of a proposed metallacyclic Fe(II) carbene intermediate. These results indicate that, with the appropriate choice of supporting ligands, it is possible to cleave and homologate carbon monoxide under mild conditions using an abundant and environmentally benign low-coordinate, first row transition metal.

## Abstract (web summary)

Metal-mediated activation of CO for C-C coupling reactions is a valuable approach to carbon monoxide valorization. Here, the authors use low-coordinate iron(II) complexes for the selective scission and homologation of CO affording unusual squaraines and iron carboxylates under mild conditions.

## Introduction

CO activation is a critical reaction in organometallic chemistry, where it can be used to form valuable organic compounds through both homogeneous and heterogeneous catalytic transformations. These include carbonylation, hydroformylation, polymerisation, hydroesterification, and syngas conversion^1–3^. Early investigations of homogeneously catalysed CO hydrogenation and oligomerisation required the use of prohibitively high temperatures and pressures, affording only simple oligomers in low yield^3^. This can be improved somewhat through the use of additives such as Lewis acids, Lewis bases, or Brønsted acids. The study of model systems such as [(η^5^-C_5_H_5_)Fe(CO)_3_]^+^, aided by the addition of reducing agents and Lewis base, allowed the fundamental steps in these processes to be elucidated^4–7^. Such electron-rich systems, however, are unable to undergo facile carbonylation; requiring borohydrides for carbonyl reduction and the use of relatively strong electrophiles to release homologated products^3^.

The literature is replete with examples of migratory insertion of CO into M−C bonds. Of particular note is the reduction of coordinated CO by insertion into M–H and M–alkyl bonds, resulting in formyl or acyl species respectively, where the formal oxidation of the ligand permits the reduction of CO^7,8^. Acyl intermediates can act as a precursor for the formation of C_2_ species and higher oligomers. Alternatively, the coupling of CO with carbenes forming η^2^-ketene complexes provides another route to C–C bond formation^9,10^. More interesting still is the chemistry reported for several zirconium complexes, where CO is not only inserted into the M−H bond, but the reductive coupling of two or more CO molecules has been reported^11–14^. Although the reductive coupling of CO remains unusual, there are a number of examples from across the periodic table, including the p-block^15,16^, d-block^12,17–26^ and f-block^27–34^.

The scission and homologation of CO to generate synthetically useful compounds under mild conditions remains a significant challenge. The C≡O bond is the strongest bond in chemistry (CO bond dissociation energy = 1076 kJ mol^‒1^)^35^ and the complete cleavage of CO requires six electrons for reduction. However, complexes of the d-block and f-block elements have been shown to form a range of compounds through mechanisms involving the complete scission of C≡O followed by homologation^12,36–44^. Previous work on C_4_ ring formation via CO activation has yielded squarates (C_4_O_4_^2‒^) from uranium complexes, which does not require the scission of C≡O bonds^27^.

The reductive coupling and cleavage of CO is unknown for low-coordinate iron species^45,46^, with early studies indicating that CO coupling was promoted by high-coordinate transition metal complexes^47^. Furthermore, previously reported reactions between transition metal *m*-terphenyl complexes and CO afforded only insertion products such as acyl complexes^48,49^ and sterically encumbered ketones^50^.

Herein, we report the complete scission of C≡O by low-coordinate Fe^II^ complexes at ambient temperature and pressure, accompanied by the formal oxidation of the terphenyl ligand, affording unusual 1,3-squaraines through C–C coupling reactions with concomitant formation of Fe^II^ carboxylate complexes and Fe(CO)_5_. These squaraine species feature broken conjugation (resulting in atypical electronic and bonding properties) and represent the first examples of C_4_ ring formation from CO involving complete cleavage of the C≡O bond.

## Results

### Reactions between iron diaryl complexes and carbon monoxide

**Fig. 1:**
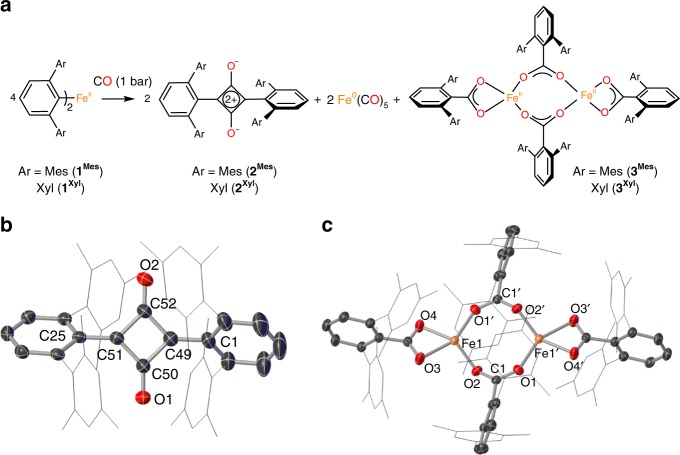
The reductive coupling and functionalisation of CO by iron(II) diaryls **1**^**Mes**^ and **1**^**Xyl**^. **a** General scheme for the reaction between CO and iron(II) diaryls **1**^**Mes**^ or **1**^**Xyl**^. **b** The molecular structure of **2**^**Mes**^ with anisotropic displacement ellipsoids set at 50% probability. Mesityl groups shown as wireframe, hydrogen atoms and one molecule of hexane solvent are omitted for clarity. **c** The molecular structure of **3**^**Mes**^ with anisotropic displacement ellipsoids set at 50% probability. Mesityl groups shown as wireframe and hydrogen atoms have been omitted for clarity. Selected bond distances (Å) and angles (°) for **3**^**Mes**^: Fe1–O1 1.9736(11), Fe1–O2 1.9357(11), Fe1–O3 2.1909(13), Fe1–O4 2.0232(12), O1–Fe1–O2 109.30(5), O3–Fe1–O4 61.77(5)

Exposure of a toluene solution of **1**^**Mes**^ ^51^ or **1**^**Xyl**^ to excess CO at ambient temperature and pressure (Fig. 1a) results in an immediate colour change from yellow to red; further stirring for 6 days (**1**^**Mes**^) or 36 h (**1**^**Xyl**^) under a CO atmosphere forms a dark red suspension. Recrystallisation from hexane at −30 °C (**1**^**Mes**^) or the layering of pentane onto a toluene solution at room temperature (**1**^**Xyl**^) affords red crystals of the squaraine molecules **2**^**Mes**^ and **2**^**Xyl**^ suitable for single crystal X-ray diffraction. The molecular structures of **2**^**Mes**^ and **2**^**Xyl**^ feature a central four-membered ring {C_4_O_2_} with *m*-terphenyl substituents bound to each side (Fig. 1b and Supplementary Fig. 2). Selected bond distances and angles for **2**^**Mes**^ and **2**^**Xyl**^ can be found in Table 1.

**Table 1:** Selected experimental (X-ray, 2^Mes^ and 2^Xyl^) and calculated (DFT optimised, **2a**) bond distances (Å) and angles (°)

	2^Mes^	2^Xyl^	2a
O1‒C50/O1‒C14	1.214(3)	1.212(2)	1.213
C52‒O2	1.218(3)		1.213
C49‒C50/C13‒C14	1.475(3)	1.4837(18)	1.472
C50‒C51	1.470(3)		1.473
C51‒C52	1.470(3)		1.473
C49‒C52	1.472(4)		1.473
C1 plane‒{C_4_O_2_}	56.38(13)	50.93(5)	47.38
C25 plane‒{C_4_O_2_}	51.71(11)		47.38

The atom labelling schemes for **2**^**Mes**^ and **2**^**Xyl**^ are displayed on the crystal structures (Fig. 1b and Supplementary Fig. 2, respectively)

In contrast to other aryl-substituted squaraines, where the aryl rings typically lie in-plane with the C_4_O_2_ ring to maximise conjugation (average dihedral angle between aryl ring and C_4_O_2_ ring = 1.24°)^52^, the aromatic rings in **2**^**Mes**^ and **2**^**Xyl**^ are twisted out of plane with the central ring [**2**^**Mes**^: 56.38(13)° and 51.71(11)°, **2**^**Xyl**^: 50.93(5)°]. This is likely due to the steric demands of the terphenyl substituents. The C–O bond lengths in these compounds are also shorter than those found in typical squaraines [**2**^**Mes**^: O1‒C50 1.214(3) Å, C52‒O2 1.218(3) Å; **2**^**Xyl**^: O1‒C14 1.212(2) Å; average squaraine C–O bond length = 1.24 Å]^52^. This is indicative of greater C–O double bond character, which is likely due to reduced conjugation with the aromatic substituents.

The Fe^II^ carboxylate complexes **3**^**Mes**^ and **3**^**Xyl**^ (Fig. 1a) are also formed in the reaction of **1**^**Mes**^ or **1**^**Xyl**^ with CO and their solid-state structures have been confirmed by X-ray crystallography. The geometry around these Fe centres (Fig. 1c and Supplementary Fig. 3) is a distorted seesaw [τ_4_ = 0.57 (**3**^**Mes**^) and 0.54 (**3**^**Xyl**^)]^53^, and results from the coordination of Fe by one terminal and two bridging carboxylate ligands in which the angles between the FeO_2_ planes defined by O1′‒Fe1‒O2 and O3‒Fe1–O4 are 46.634(3)° and 40.94(13)° for **3**^**Mes**^ and **3**^**Xyl**^, respectively. When one considers the balanced reaction scheme (Fig. 1a), it is important to mention that the formal oxidation of the ligand is essential for the reduction of CO. In the overall reaction, there is a net reduction of iron by four electrons and a net reduction of CO by four electrons, giving a total net reduction of eight electrons from the oxidation of the aryl ligands. The reaction between **1**^**Mes**^ and CO was found to be near-quantitative, as determined by NMR integration against an internal standard (see Supplementary Methods). However, isolated yields were low due to difficulties in separating **2**^**Mes**^ from **3**^**Mes**^, as the compounds have similar solubility. Analogous attempts to quantify the reaction between **1**^**Xyl**^ and CO by NMR spectroscopy were hampered by the precipitation of **2**^**Xyl**^**/3**^**Xyl**^ mixtures (see Supplementary Methods).

Squaraine **2**^**Mes**^ has been characterised by ^1^H and ^13^C{^1^H} NMR, IR and UV/Vis spectroscopies and by mass spectrometry. The ^13^C{^1^H} NMR spectrum [C_6_D_6_ solution, Supplementary Fig. 5(a)] contains peaks at *δ*_C_ 269.7 and 177.3 ppm, corresponding to the carbonyl (O*C*C) and the terphenyl-bound carbons in the C_4_O_2_ ring, respectively. The assignment of the chemical shift at 269.7 ppm to the remarkably deshielded carbonyl (O*C*C) of **2**^**Mes**^ is confirmed by ^1^H,^13^C-HMBC NMR spectroscopy [Supplementary Fig. 5(b)]. This, again, most likely results from the steric demands of the pendant terphenyl groups forcing the aryl ring to twist out-of-plane with the C_4_O_2_ ring, which results in less charge delocalisation. In addition, most squaraines possess a donor–acceptor–donor (D–A–D) configuration where charge is delocalised into the C_4_O_2_ ring^54–57^, a feature which is absent here. For example, squaraines with *N*,*N*-diarylanilino substituents display ^13^C{^1^H} NMR peaks for the C_4_O_2_ ring in the range of 160 to 185 ppm^58^. Similarly, the solution IR spectrum of **2**^**Mes**^ in toluene possesses a C–O stretch at 1673 cm^−1^ (Supplementary Fig. 12), which is at a higher frequency than typical squaraines (range 1594–1633 cm^‒1^) ^54,56,59^.

To gain insight into the structures of **2**^**Mes**^ and **2**^**Xyl**^, gas-phase DFT geometry optimisations were performed on a model compound of **2**^**Mes**^ and **2**^**Xyl**^ (**2a**), in which the flanking mesityl and xylyl substituents were replaced by phenyl groups. Calculations for **2a** give geometrical parameters (Table 1) that are in good agreement with those of the experimentally determined structures of **2**^**Mes**^ and **2**^**Xyl**^. Additionally, **2a** features a non-planar core with a dihedral angle between C_4_O_2_ ring and the aryl substituent of 47.38°, slightly smaller than those observed in **2**^**Mes**^ and **2**^**Xyl**^ [**2**^**Mes**^: 56.38(13)° and 51.71(11)°, **2**^**Xyl**^: 50.93(5)°]. Squaraines have previously been isolated in the singlet ground state^60^ and RASSCF calculations^61^ indicate that closed-shell singlet is the predominant electronic configuration for **2a** (see Supplementary Methods). The calculated C‒O stretching frequency for **2a** is 1720 cm^−1^ (scaled by 0.95), which is higher than that found experimentally [1673 cm^−1^ (**2**^**Mes**^) and 1695 cm^−1^ (**2**^**Xyl**^)]. This is not unexpected, as it has been documented that calculations which assume a harmonic approximation may overestimate IR stretching frequencies^38^.

### Labelling studies and mechanistic investigations

**Fig. 2:**
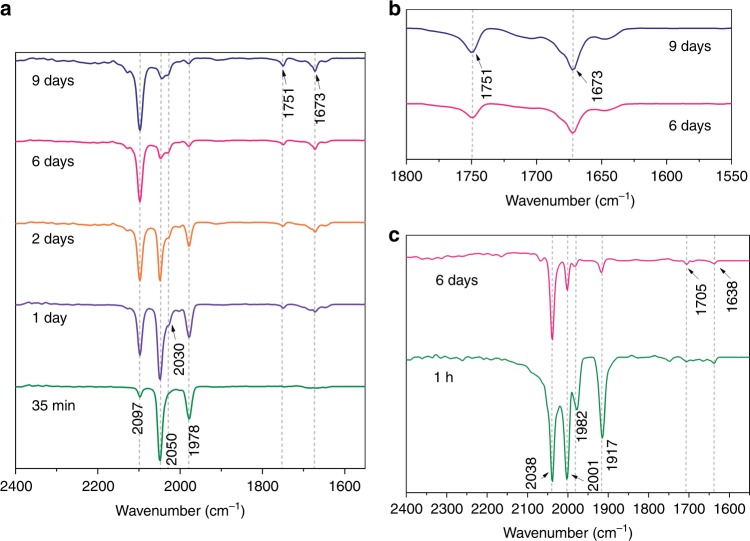
IR spectroscopic monitoring of the reaction between **1**^**Mes**^ and carbon monoxide. **a** IR spectra in toluene at room temperature obtained from the reaction of **1**^**Mes**^ with CO; an aliquot was taken from the reaction mixture at the indicated times, dried under vacuum for ca. 1 h and dissolved in toluene. **b** Zoom of **a** between 1800 and 1550 cm^–1^; spectra recorded at 6 days and 9 days. **c** IR spectra in toluene at room temperature obtained from the reaction of **1**^**Mes**^ with ^13^CO; an aliquot was taken from reaction mixture at the indicated times, dried under vacuum for ca. 1 h and dissolved in toluene

Reactions between **1**^**Mes**^ or **1**^**Xyl**^ and ^13^CO show that the squaraine molecules (**2**^**Mes**^ and **2**^**Xyl**^) incorporate four C atoms [C49 to C52 (**2**^**Mes**^) and C13 to C14 (**2**^**Xyl**^)] from CO into the central C_4_O_2_ ring. This requires complete C≡O bond cleavage and the formation of new C–C bonds, which is significant as the reactions between CO and **1**^**Mes**^ or **1**^**Xyl**^ take place under ambient conditions in the absence of an external reducing agent. The reaction between **1**^**Mes**^ and ^13^CO generates **2**^**Mes**^**-**^**13**^**C**, where the ^13^C{^1^H} NMR spectrum displays triplet resonances for the central ^13^C_4_O_2_ ring [*δ*_C_ 269.7 ppm (t, ^1^*J*(C,C) = 45 Hz, *C*O), 177.3 ppm (t, ^1^*J*(C,C) = 45 Hz, *C*CO)]. Furthermore, ^13^C,^13^C-COSY NMR (Supplementary Fig. 6) shows a correlation between these C atoms, providing further evidence that the four carbon atoms of the central ^13^C_4_O_2_ ring must originate from ^13^CO. The solution IR spectrum of **2**^**Mes**^**-**^**13**^**C** displays an absorption at 1638 cm^−1^ (Fig. 2c). DFT calculations of **2a-**^**13**^**C** predict a vibrational frequency (1675 cm^‒1^) that is 37 cm^−1^ higher than that determined experimentally (Supplementary Table 3). However, the ratio of the calculated frequencies (^13^C/^12^C = 0.974), compares well with that of the experimental values (0.979). Isolated samples of **2**^**Mes**^**-**^**13**^**C** contain a minor by-product that is observed as two doublets in the ^13^C{^1^H} NMR spectrum [*δ*_C_ 193.6 (d, ^1^*J*(C,C) = 108 Hz, C=C=O), 24.8 (d, ^1^*J*(C,C) = 108 Hz, C=C=O), see Supplementary Fig. 7], which are attributed to a ketene- or ketenyl-type intermediate in the formation of **2**^**Mes**^ and **2**^**Xyl**^ ^37,62^.

To gain insight into the reaction between **1**^**Mes**^ and CO, the progress of the reaction was monitored by IR (Fig. 2a, b) and NMR spectroscopies. Initially, three new IR bands appeared 35 min after the addition of CO to the reaction mixture (*ν* = 2097, 2050, 1978 cm^−1^). The band at 2097 cm^−1^ increased in intensity whilst the bands at 2050 and 1978 cm^−1^ decreased in intensity over the course of the reaction. The bands at 2050 and 1978 cm^−1^ are most likely associated with terminal Fe carbonyls^63^ and occur in a similar range to those of the Fe^II^ carbonyl species, (η^2^-2,6-Dipp_2_C_6_H_3_CO)_2_Fe(CO)_2_, (*ν* = 2000, 1944 cm^−1^)^48^, whilst the band at 2097 cm^−1^ is consistent with a ketene or ketenyl species (C=C=O), which appear in the range 2080 to 2250 cm^−1^ ^62^. The reaction between **1**^**Mes**^ and ^13^CO was also monitored by IR spectroscopy (Fig. 2c) and features bands between 1638–2038 cm^−1^, all of which are shifted with respect to those observed in the analogous reaction between **1**^**Mes**^ and CO (Supplementary Table 1). Monitoring the reaction by ^1^H and ^13^C{^1^H} NMR spectroscopy in C_6_D_6_ shows the disappearance of the peaks associated with **1**^**Mes**^ immediately after the addition of CO, followed by the appearance of **2**^**Mes**^ and paramagnetically shifted peaks after 19 h. After 30 h crystals formed, which were determined to be a mixture of **2**^**Mes**^ and **3**^**Mes**^ by X-ray diffraction. The formation of Fe(CO)_5_ was confirmed in the reaction between **1**^**Mes**^ or **1**^**Xyl**^ and CO by IR and NMR spectroscopy, with bands at 2024 and 1999 cm^−1^ (Supplementary Fig. 15)^64^, and a singlet at 211 ppm in the ^13^C{^1^H} NMR spectra.

**Fig. 3:**
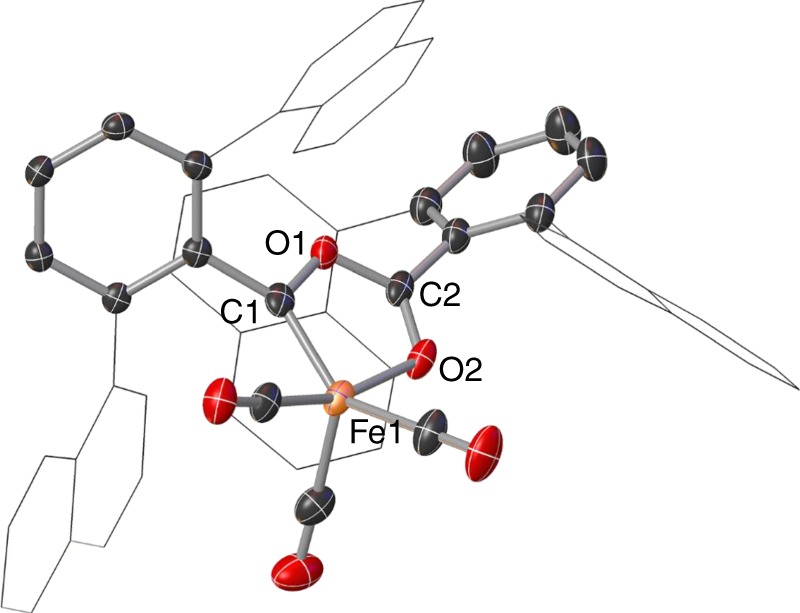
Molecular structure of **4**. Structure of **4** with anisotropic displacement ellipsoids set at 50% probability. Naphthyl groups shown as wireframe, hydrogen atoms and one molecule of Et_2_O solvent have been omitted for clarity. Selected bond distances (Å) for **4**: Fe1–C1 1.840(3), Fe1–O2 1.9572(18), Fe1–C3 1.830(3), Fe1–C4 1.753(3), Fe1–C5 1.849(3), C1–Fe1–O2 81.16(9)

To investigate further the influence of the aryl substituents on the reaction of *m*-terphenyl iron(II) complexes with CO, a solution of (2,6-Naph_2_C_6_H_3_)_2_Fe(THF) (**1**^**Naph**^; Naph = 1-C_10_H_7_) in Et_2_O was exposed to CO. After stirring at room temperature overnight, the reaction was worked up to afford red crystals of the metallacyclic Fe^II^ carbene (CO)_3_Fe[C(2,6-Naph_2_C_6_H_3_)OC(O)(2,6-Naph_2_C_6_H_3_)] (**4**) that were suitable for X-ray diffraction measurements (Fig. 3). The solid-state structure of **4** exhibits an Fe^II^ centre in a distorted square-pyramidal environment (τ_5_ = 0.37). Compound **4** displays complex (see Supplementary Discussion and Supplementary Fig. 8) diamagnetic ^1^H and ^13^C{^1^H} NMR spectra (Supplementary Figs. 9 and 10) and could represent an isolable analogue of an intermediate species in the reaction of **1**^**Mes**^**/1**^**Xyl**^ with CO. Treating compound **4** with excess CO failed to yield any further reaction, even after prolonged heating (80 °C for 14 h, see Supplementary Methods).

**Fig. 4:**
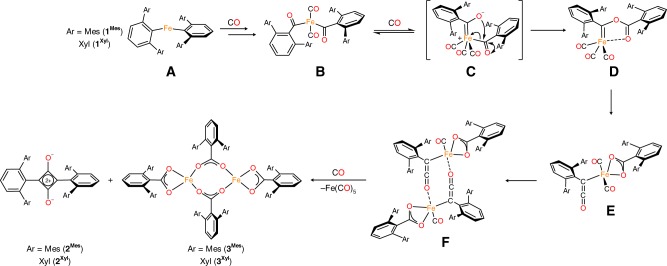
Proposed pathway for the conversion of the iron(II) diaryls **1**^**Mes**^, **1**^**Xyl**^ and **1**^**Naph**^ to **2**–**4**. Proposed mechanism for the reaction between **1**^**Mes**^, **1**^**Xyl**^, and **1**^**Naph**^ and CO forming squaraines **2**^**Mes**^ and **2**^**Xyl**^, carboxylate complexes **3**^**Mes**^ and **3**^**Xyl**^ and carbene complex **4**. In the case of Ar = naphthyl, **D** = complex **4**

It is possible to propose a pathway for the formation of the squaraines (**2**^**Mes**^ and **2**^**Xyl**^) and Fe carboxylates (**3**^**Mes**^ and **3**^**Xyl**^) from **1**^**Mes**^ and **1**^**Xyl**^ (Fig. 4) that is consistent with all spectroscopic data and the observation of compound **4**. The proposed mechanism also accounts for the observation (from ^13^C labelling) that all carbons in the C_4_O_2_ ring derive from CO.

First, CO coordinates to the Fe^II^ centre in the diaryl complexes **A**, followed by 1,1-migratory insertion of CO into the Fe−C σ-bonds of the two *m*-terphenyl ligands to form an acyl intermediate of the type **B**. A similar Fe^II^ acyl complex has previously been observed in the reaction of (2,6-Dipp_2_C_6_H_3_)_2_Fe with CO^48,49^. Compound **B** then undergoes intramolecular C−O bond formation via keto-carbene tautomerisation to the carbene intermediate **C**^65,66^. Attack of the carbonyl carbon by the CO^−^ unit forms the Fe carbene intermediate (**D**)^65,66^, which is analogous to the Fe carbene complex **4**. Species **D** may then react with CO^44,62,67,68^, to produce a ketenyl complex, with concomitant CO bond scission between the carbene C and carboxylate O to form intermediate **E**. Species **E** then dimerises to form iron ketenyl carboxylate complex **F**, similar to those observed in the reaction of CO with low valent organolanthanide complexes^38,40^. The coordinated ketene units may then dimerise in a [2 + 2] cycloaddition reaction affording **2**^**Mes**^/**2**^**Xyl**^ in an analogous manner to that for uncoordinated ketenes, which dimerise to form 1,3-cyclobutanediones^62^. The concomitant dimerisation of the Fe-carboxylate units in **F** form the diiron carboxylate complexes **3**^**Mes**^/**3**^**Xyl**^ and liberate Fe(CO)_5_.

Given the disparate reactivities of **1**^**Naph**^ and **1**^**Xyl**^/**1**^**Mes**^ on treatment with CO, the mechanism proposed in Fig. 4 was probed by DFT, focusing on the conversion of **D** to **E** (see Supplementary Methods). For the xylyl substituted complex, the barrier to reaction (Δ^‡^*G*°) is +14.6 kcal mol^‒1^, with a favourable Δ_r_*G*° of ‒15.6 kcal mol^‒1^. However, formation of the naphthyl substituted **E** has a larger energetic barrier (Δ^‡^*G*° = 22.8 kcal mol^‒1^) and is thermodynamically unfavourable (Δ_r_*G*° = +19.6 kcal mol^‒1^). This striking difference is attributed to greater steric repulsion in **E**^**Naph**^, where the CO ligands and ketene clash with the large naphthyl flanking groups (Supplementary Fig. 22). This result helps rationalise why **4** is an isolable complex, while the corresponding carbene is not observed in the reactions of **1**^**Mes**^ and **1**^**Xyl**^ with CO, and highlights how the reactivity of these systems is heavily dependent on the nature of the flanking aryl groups.

This reactivity is rather remarkable when compared with other open shell iron(II) hydrocarbyls^45,46^ and transition metal terphenyls^48–50^ which, although they undergo carbon monoxide coordination and migration, do not cleave C≡O bonds. Presumably this is due to factors such as the overall ligand field strength, complex geometry, and steric effects, which we have shown (through theoretical calculations) are heavily dependent on the flanking aryl groups of the *m*-terphenyl ligands. The importance of steric effects in CO activation has been demonstrated previously^19,69^. Furthermore, our system contrasts with electron-rich systems such as the [(η^5^-C_5_H_5_)Fe(CO)_3_]^+^ model complex, which do not readily undergo carbonyl reduction and require the use of electrophiles to release homologated products^3^.

### Electrochemical investigations of squaraines

The electrochemical properties of **2**^**Mes**^ were investigated by cyclic voltammetry, which revealed a reversible reduction process at *E*_1/2_ = ‒0.79 V vs Fc^+^/Fc (Supplementary Fig. 13). This is significantly more anodic than typical squaraines, which possess reduction processes in the range of ‒1.40 to ‒0.98 V vs Fc^+^/Fc^55,58^. The reduction process is localised principally on the C_4_O_2_ ring in **2**^**Mes**^ (vide infra) and the difference in reduction potential is consistent with this moiety being electron deficient due to decreased conjugation with the aromatic rings. In addition, **2**^**Mes**^ displays an irreversible oxidation process at *E*_p_^a^ = + 0.48 V vs Fc^+^/Fc (Supplementary Fig. 14).

**Fig. 5:**
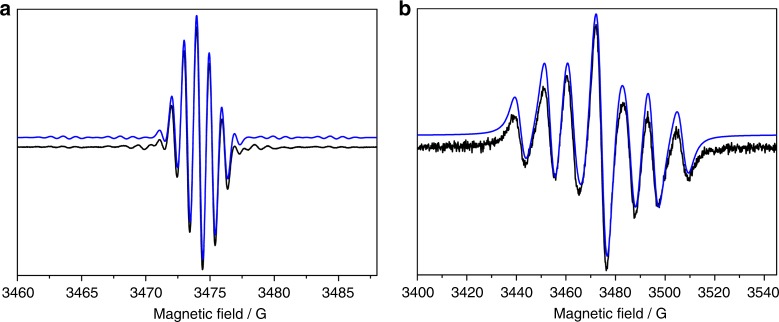
Spectroscopic investigations of **2**^**Mes**^ with Cp_2_Co in CH_2_Cl_2_ at room temperature. **a** Experimental X-band EPR spectrum of **2**^**Mes•‒**^ (black trace) recorded as a fluid solution in CH_2_Cl_2_ at room temperature. The simulated spectrum is given in blue and parameters used for the simulation are listed in Supplementary Table 2. **b** Experimental X-band EPR spectrum of **2**^**Mes•‒**^**-**^**13**^**C** (black trace) recorded as a fluid solution in CH_2_Cl_2_ at room temperature. The simulated spectrum is given in blue and parameters used for the simulation are listed in Supplementary Table 2

Monoanionic **2**^**Mes•‒**^ and **2**^**Mes•‒**^**-**^**13**^**C** were prepared by the addition of Cp_2_Co (Cp = η^5^-C_5_H_5_) to a solution of **2**^**Mes**^ or **2**^**Mes**^**-**^**13**^**C** in CH_2_Cl_2_. The experimental X-band EPR spectra, along with the simulated spectra, are shown in Fig. 5. The spectra show hyperfine couplings to ^1^H and ^13^C centres and may be simulated using the spin Hamiltonian parameters shown in Supplementary Table 2. The room temperature X-band EPR spectrum of **2**^**Xyl•−**^ in CH_2_Cl_2_ solution (generated from the addition of Cp_2_Co to **2**^**Xyl**^) is shown in Supplementary Fig. 19, with simulation parameters in Supplementary Table 2. Unrestricted DFT calculations of the hyperfine coupling constants of **2a**^**•‒**^ (Supplementary Table 4) show a close correspondence with those derived from the EPR simulations of **2**^**Mes•‒**^ and are consistent with a spin density distribution (Supplementary Fig. 21) that lies across the central {(C_6_H_3_)_2_C_4_O_2_} core with little delocalisation onto the flanking aryl substituents.

## Discussion

Two-coordinate iron(II) diaryl complexes promote the reductive cleavage and homologation of CO to yield {C_4_O_2_} species at room temperature and atmospheric pressure, converting C_1_ feedstocks into useful organic molecules through a single first-row transition metal site. Unlike past work on C_4_ ring formation through homologation, these reactions proceed via C≡O bond cleavage, and afford unusual squaraine species featuring broken conjugation. The observation of well-defined intermediates in this reaction, in addition to evidence from spectroscopy and isotopic labelling, allows us to rationalise the CO activation processes and propose a reasonable mechanism that is in-line with experimental observations. Furthermore, this investigation provides additional insight into CO activation by transition metals, which is of relevance to a vast array of industrially important catalytic processes.

## Methods

For synthetic details, analytical data, full descriptions of the methods and details of the computational calculations contained in this paper see Supplementary Information. For full synthetic procedures and analytical data for the compounds herein see Supplementary Methods. For a view of the single crystal X-ray structure for compounds **1**^**Xyl**^, **2**^**Xyl**^, **3**^**Xyl**^, **1**^**Naph**^ see Supplementary Figs. 1–4, respectively. For selected NMR spectra of the compounds in this article see Supplementary Figs. 5–7 and 9–11. Cyclic voltammograms of **2**^**Mes**^ are found in the Supplementary Figs. 13 and 14. Additional IR spectra and reaction monitoring figures are found in Supplementary Figs 12 and 15–18. EPR parameters for the experimental and simulated EPR spectra for **2**^**Mes·‒**^, **2**^**Mes·‒13C**^ and **2**^**Xyl·‒**^ are given in Supplementary Table 2. Computational methods are outlined in the Supplementary Methods. Geometry-optimised co-ordinates of all computed structures are given in Supplementary Tables 5–13.

## Electronic supplementary material

Supplementary Information

## Data Availability

X-ray crystallographic data for compounds **1**^**Xyl**^-**4** are available free of charge from The Cambridge Crystallographic Data Centre (CCDC 1589889-1589895) via https://www.ccdc.cam.ac.uk/data_request/cif. All other data are available from the authors upon reasonable request.
