# Supplementary material for: Selective reduction and homologation of carbon monoxide by organometallic iron complexes
Source: Nat Commun. 2018 Sep 14;9:3757. doi: 10.1038/s41467-018-06242-w (PMC6138626; doi:10.1038/s41467-018-06242-w)
Supplement: Supplementary file 1 — Supplementary Information [file 41467_2018_6242_MOESM1_ESM.pdf]

Supporting Information for:

Selective Reduction and Homologation of Carbon Monoxide by Organometallic Iron Complexes

Sharpe et al.

Supplementary Methods 5

General procedures and starting materials. 5

Synthetic Methods 6

(2,6-(2,6-Xyl)$_2$C$_6$H$_3$)$_2$Fe (**1**$^{\mathbf{Xyl}}$) 6

1,3-(2,6-Mes$_2$C$_6$H$_3$)$_2$C$_4$O$_2$ (**2**$^{\mathbf{Mes}}$) 6

1,3-(2,6-Mes$_2$C$_6$H$_3$)$_2$^{13}C$_4$O$_2$ (**2**$^{\mathbf{Mes\text{-}^{13}}}$**C**) 7

[(2,6-Mes$_2$C$_6$H$_3$CO$_2$)Fe(μ-CO$_2$C$_6$H$_3$-2,6-(2,6-Mes)$_2$]$_2$ (**3**$^{\mathbf{Mes}}$) 7

Reaction of (2,6-(2,6-Xyl)$_2$C$_6$H$_3$)$_2$Fe (**1**$^{\mathbf{Xyl}}$) with CO 8

(2,6-Naph$_2$C$_6$H$_3$)$_2$Fe(THF) (**1**$^{\mathbf{Naph}}$) 8

(CO)$_3$Fe[C(2,6-Naph$_2$C$_6$H$_3$)OC(O)(2,6-Naph$_2$C$_6$H$_3$)]·Et$_2$O (**4**) 9

Reaction Monitoring *via* IR Spectroscopy 11

Reaction Monitoring *via* NMR Spectroscopy 12

Reaction of **1**$^{\mathbf{Mes}}$ 12

Reaction of **1**$^{\mathbf{Xyl}}$ 12

Further Reaction of Compound **4** with CO 12

EPR Spectroscopy 13

Crystallographic Methods 14

Crystal data for (2,6-(2,6-Xyl)$_2$C$_6$H$_3$)$_2$Fe (**1**$^{\mathbf{Xyl}}$) 14

Crystal data for 1,3-(2,6-Mes$_2$C$_6$H$_3$)$_2$C$_4$O$_2$ (**2**$^{\mathbf{Mes}}$) 14

Crystal data for 1,3-(2,6-Xyl$_2$C$_6$H$_3$)$_2$C$_4$O$_2$ (**2**$^{\mathbf{Xyl}}$) 15

Crystal data for [(2,6-Mes$_2$C$_6$H$_3$CO$_2$)Fe(μ-CO$_2$C$_6$H$_3$-2,6-(2,6-Mes)$_2$]$_2$ (**3**$^{\mathbf{Mes}}$) 15

Crystal data for [(2,6-Xyl$_2$C$_6$H$_3$CO$_2$)Fe(μ-CO$_2$C$_6$H$_3$-2,6-(2,6-Xyl)$_2$]$_2$ (**3**$^{\mathbf{Xyl}}$) 15

Crystal data for (2,6-Naph$_2$C$_6$H$_3$)$_2$Fe(THF) (**1**$^{\mathbf{Naph}}$): 15

Crystal data for (CO)$_3$Fe[C(2,6-Naph$_2$C$_6$H$_3$)OC(O)(2,6-Naph$_2$C$_6$H$_3$)] (**4**): 15

Computational Methods 17

Computational Analysis of Squaraine 17

Calculation of hyperfine coupling constants 17

Computational Analysis of Proposed Reaction Pathway 18

Supplementary Discussion **19**

NMR spectra of Compound **4** 19

Supplementary Figures **20**

Crystal Structure Figures 20

Supplementary Figure 1: Crystal Structure of $\mathbf{1^{Xyl}}$ 20

Supplementary Figure 2: Crystal Structure of $\mathbf{2^{Xyl}}$ 20

Supplementary Figure 3: Crystal Structure of $\mathbf{3^{Xyl}}$ 21

Supplementary Figure 4: Crystal Structure of $\mathbf{1^{Naph}}$ 22

NMR Spectra and Related Figures 23

Supplementary Figure 5: Selected NMR Spectra of $\mathbf{2^{Mes}}$ 23

Supplementary Figure 6: ^{13}C,^{13}C COSY NMR Spectra of $\mathbf{2^{Mes}}$-$\mathbf{^{13}C}$ 23

Supplementary Figure 7: Selected region of ^{13}C{^{1}H} NMR Spectra of $\mathbf{2^{Mes}}$-$\mathbf{^{13}C}$ 24

Supplementary Figure 8: Structure of compound **4** and possible *syn*- and *anti*-conformers 24

Supplementary Figure 9: ^{1}H NMR Spectrum of **4** in C_6D_6 25

Supplementary Figure 10: ^{13}C NMR Spectrum of **4** in C_6D_6 26

Supplementary Figure 11: Variable Temperature ^{1}H NMR spectra of **4** 27

IR and EPR Spectroscopy Figures 28

Supplementary Figure 12: Solution IR Spectra of $\mathbf{2^{Mes}}$ 28

Supplementary Figure 13: Cyclic Voltammograms of $\mathbf{2^{Mes}}$ (Reduction) 28

Supplementary Figure 14: Cyclic Voltammograms of $\mathbf{2^{Mes}}$ (Oxidation) 29

Supplementary Figure 15: IR Spectra from the reaction of $\mathbf{1^{Mes}}$ with CO (toluene) 29

Supplementary Figure 16: IR Spectra from the reaction of $\mathbf{1^{Mes}}$ with CO (CH_2Cl_2) 30

Supplementary Figure 17: IR Spectra from the reaction of $\mathbf{1^{Xyl}}$ with CO (toluene) 31

Supplementary Figure 18: IR Spectra from the reaction of $\mathbf{1^{Xyl}}$ with CO (CH_2Cl_2) 32

Supplementary Figure 19: EPR Spectrum of $\mathbf{2^{Xyl\bullet-}}$ 33

Computational Figures 34

Supplementary Figure 20: Structure of **2b** 34

Supplementary Figure 21: Computed structure and spin density for **2a**$^{\bullet-}$ 34

Supplementary Figure 22: Optimised geometries for **D**^{Naph}, **E**^{Naph}, **D**^{Xyl}, and **E**^{Xyl} ...35

Supplementary Tables.. 36

Supplementary Table 1: Bands (cm^{-1}) and isotopic shifts (cm^{-1}) observed in the reaction between **1**^{Mes} and CO or ^{13}CO..36

Supplementary Table 2: Parameters for the experimental and simulated EPR spectra for **2**$^{Mes\bullet-}$, **2**$^{Mes\bullet-}$**-**^{13}**C** and **2**$^{Xyl\bullet-}$...36

Supplementary Table 3: Scaled calculated harmonic frequencies (cm^{-1}), from the DFT calculations. A scaling factor of 0.95 has been applied. 37

Supplementary Table 4: Calculated isotropic hyperfine couplings from DFT calculations on **2a**$^{\bullet-}$...41

Supplementary Table 5: Geometry optimised coordinates of **2a**.............................41

Supplementary Table 6: Geometry optimised coordinates of **2b** 43

Supplementary Table 7: Geometry optimised coordinates of **2a**$^{\bullet-}$ 44

Supplementary Table 8: Geometry optimised coordinates of **D**^{Naph} (compound **4**) .46

Supplementary Table 9: Geometry optimised coordinates of **E**^{Naph} 48

Supplementary Table 10: Geometry optimised coordinates of **D**^{Xyl}.........................50

Supplementary Table 11: Geometry optimised coordinates of **E**^{Xyl}.........................52

Supplementary Table 12: Geometry optimised coordinates for the transition state calculation for **D**^{Xyl} → **E**^{Xyl} 54

Supplementary Table 13. Geometry optimised coordinates for the transition state calculation for **D**^{Naph} → **E**^{Naph}...56

Supplementary References.. 58

Supplementary Methods

General procedures and starting materials.

All reactions and manipulations were performed by using standard Schlenk line and glovebox equipment under an atmosphere of purified argon or nitrogen. *Iso*-hexane (contains <5% *n*-hexane) was dried by passing through a column of activated 4 Å molecular sieves. THF, diethyl ether, toluene and CH_2Cl_2 were freshly distilled over sodium benzophenone ketyl (THF), molten NaK alloy (diethyl ether), molten potassium (toluene) or CaH_2 (CH_2Cl_2) under nitrogen. All solvents were degassed *in vacuo* and stored over a potassium mirror (*iso*-hexane, diethyl ether, toluene) or activated 4 Å molecular sieves (THF, CH_2Cl_2) prior to use. Benzene-d_6 was dried over potassium and degassed with three freeze/pump/thaw cycles prior to use. 1,1,2,2-tetrachloroethane-d_2 was dried over molecular sieves (3 Å) and degassed with three freeze/pump/thaw cycles prior to use. NMR spectroscopic data were recorded using a Bruker DPX400, AV400, AV(III)400, AV(III)400HD or AV(III)500 spectrometer. Chemical shifts are quoted in ppm relative to neat TMS (^{1}H, ^{13}C{^{1}H}). Residual solvent signals were used as internal references for ^{1}H and ^{13}C{^{1}H} NMR measurements, and ^{1}H, ^{13}C and assignments were confirmed with the use of ^{1}H,^{1}H, ^{1}H,^{13}C and ^{13}C,^{13}C correlation experiments where necessary. (2,6-$Mes_2C_6H_3)_2Fe$[1] (**1**$^{\mathbf{Mes}}$), [2,6-$Xyl_2C_6H_3Li]_2$[2] and [$Naph_2C_6H_3Li]_2$[3] were prepared following the procedures described in the literature. CO gas (Carbon Monoxide CP Grade N3.0) was supplied by BOC; N3.0 gas has a minimum purity of 99.9%. ^{13}CO (^{13}C, 99%) was supplied by CK Isotopes and used as received. Mass spectra were measured by the departmental service at the University of Nottingham or at the EPSRC UK National Mass Spectrometry Facility at Swansea University. IR absorption spectroscopy was recorded in solutions in dry degassed solvents in a cell with KBr windows on a Bruker Alpha FTIR instrument. Continuous wave X-band EPR spectra were carried in a Young's tap modified Wilmad quartz EPR tube and recorded using a Bruker EMX spectrometer at room temperature. The simulations of CW EPR spectra were performed using the Bruker WINEPR SimFonia package. Cyclic voltammetry (CV) measurements were carried out using an Autolab PGSTAT320N potentiostat and performed using a three-electrode system in a single compartment cell containing a glassy carbon working electrode, a Pt wire secondary electrode and a saturated calomel reference electrode, chemically isolated from the test solution *via* a bridge tube containing electrolyte solution and fitted with a porous vycor frit. The concentration of

solutions were 1 mM for test compound and 0.4 M for the supporting electrolyte [$^{n}Bu_4N$][BF_4] in CH_2Cl_2. CV was performed under an argon atmosphere. Redox potentials are referenced relative to the Fc/Fc^+ couple by an internal calibration. UV/visible absorption samples were prepared as solutions of known concentrations under an inert atmosphere. Spectra were obtained using a Young's tap modified 10 mm quartz cell using a Perkin Elmer Lambda 5 or Lambda 750 spectrophotometer over a wavelength range of 200-1100 nm with 1 nm data spacing. Elemental microanalysis was performed by Mr Stephen Boyer at the Microanalysis Service, London Metropolitan University, UK.

Synthetic Methods

(2,6-(2,6-Xyl)$_2$C$_6$H$_3$)$_2$Fe (1^{Xyl})

To a mixture of [2,6-Xyl$_2$C$_6$H$_3$Li]$_2$ (500 mg, 0.855 mmol) and FeCl$_2$(THF)$_{1.5}$ (201 mg, 0.855 mmol), were added toluene (20 mL) and THF (2 mL) resulting in a clear dark yellow solution which was stirred at room temperature for 16 h. The solvent was removed *in vacuo* resulting in a pale-yellow solid which was dried for 4 h. The product was extracted into toluene (2 × 10 mL) and the solution was concentrated to *ca.* 10 mL *in vacuo* and stored at −30 °C for 2 days resulted in the precipitation of a yellow crystalline solid (280.2 mg, 52%). ^{1}H NMR (400 MHz, C_6D_6, 25 ºC): δ 32.49 (s, br, $\Delta\nu_{1/2}$ = 304 Hz), 2.05 (s, br, $\Delta\nu_{1/2}$ = 52 Hz), −35.26 (s, br, $\Delta\nu_{1/2}$ = 1220 Hz), −54.62 (s, br, $\Delta\nu_{1/2}$ = 244 Hz), −59.32 (s, br, $\Delta\nu_{1/2}$ = 188 Hz). μ_{eff} (Evans, C_6D_6, 25 ºC): 5.08 μ_B. Elemental analysis $C_{44}H_{42}Fe$: calcd. C 84.33, H 6.76; found C 84.16, H 6.65. MS(EI) *m/z* = 626.3 [M]$^+$ (11.1%), fragment ion peak at *m/z* 341.1 [2,6-(2,6-Xyl)$_2$C$_6$H$_3$Fe]$^+$ (5.0%). IR (Nujol mull): ν/cm^{-1} = 1959 (w), 1925 (w), 1574 (w), 1547 (m), 1305 (m), 1260 (m), 1244 (m), 1161 (m), 1094 (m), 1071 (m), 1027 (m), 984 (w), 801 (s), 766 (s), 738 (s), 723 (s), 698 (w), 687 (w), 550 (w). UV/Vis (THF) $\lambda_{max/nm}$ (ε/dm^3 mol^{-1} cm^{-1}): 414 (418.6), 381 (768.6), 365 (1025.5), 356 (1156.9).

1,3-(2,6-Mes$_2$C$_6$H$_3$)$_2$C$_4$O$_2$ (2^{Mes})

In an Young's flask, a stirred solution of **1^{Mes}** (101 mg, 0.147 mmol) in toluene (5 mL) was exposed to an atmosphere of dry CO at room temperature whereupon an immediate colour change from yellow to red occurred. The reaction mixture was stirred for 6 days after which the solvent was removed *in vacuo* and the product extracted into hexane (20 mL), the solution was concentrated *in vacuo* and kept at −30 °C. This resulted in the precipitation

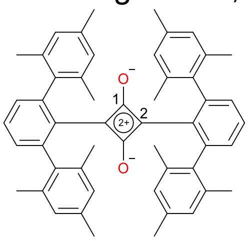

of red needle crystals suitable for X-ray diffraction. 1,3-(2,6-$Mes_2C_6H_3)_2C_4O_2$ (**2^{Mes}**) (13.0 mg, 24%). ^{1}H NMR (500 MHz, C_6D_6, 25 ºC): δ 7.01 (t, ^3J(H,H) = 7.7 Hz, 2H, $C_6H_3{}^{p}$), 6.86 (s, 8H, C*H*-Mes), 6.79 (d, ^3J(H,H) = 7.7 Hz, 4H, $C_6H_3{}^{m}$), 2.19 (s, 12H, C$H_3{}^{p}$-Mes), 1.98 (s, 24H, C$H_3{}^{o}$-Mes). ^{13}C{^{1}H} (126 MHz, C_6D_6, 25 ºC) δ = 269.7 (t, ^1J(C,C) = 45 Hz, *C*O), 177.3 (t, ^1J(C,C) = 45 Hz, C^2), 141.1 ($C_6H_3{}^{o}$), 137.0 (Mes^{p}), 136.8 (Mes^{ipso}), 136.6 (Mes^{o}), 133.7 ($C_6H_3{}^{p}$), 132.2 ($C_6H_3{}^{ipso}$), 129.3 ($C_6H_3{}^{m}$), 128.9 (Mes^{m}), 21.3 (*C*$H_3{}^{p}$-Mes), 21.0 (*C*$H_3{}^{o}$-Mes). HRMS(ES+) *m/z:* calcd. for $C_{52}H_{51}O_2$ [M+H]$^+$ 707.3883, found: 707.3871 (err [ppm] = 1.80). UV/Vis (CH_2Cl_2) at λ_{max}/nm (ε/dm^3 mol^{-1} cm^{-3}): strong absorption between 200 and 300 nm (>8000), 480 (8904), 515 (9329), 742 (3110). IR (Toluene) *v*/cm^{-1}: 1673 (C–O), IR (CH_2Cl_2) *v*/cm^{-1}: 1668 (C–O).

1,3-(2,6-$Mes_2C_6H_3)_2{}^{13}C_4O_2$ (2^{Mes}-^{13}C)

In a Young's flask, a stirred solution of **1^{Mes}** (40.3 mg, 0.0586 mmol) in toluene (8 mL) was exposed to an atmosphere of dry ^{13}CO at room temperature whereupon an immediate colour change from yellow to red solution occurred. The reaction mixture was stirred for a further 6 days after which the solvent was removed *in vacuo* and the product extracted into hexane (20 mL), the solution was concentrated *in vacuo* and kept at −30 °C. This resulted in the precipitation of red needles of 1,3-(2,6-$Mes_2C_6H_3)_2{}^{13}C_4O_2$ (**2^{Mes}-^{13}C**). ^{1}H NMR (500 MHz, C_6D_6, 25 ºC) δ = 7.01 (t, ^3J(H,H) = 7.7 Hz, 2H, $C_6H_3{}^{p}$), 6.86 (s, 8H, C*H*-Mes), 6.79 (d, ^3J(H,H) = 7.7 Hz, 4H, $C_6H_3{}^{m}$), 2.19 (s, 12H, C$H_3{}^{p}$-Mes), 1.98 (s, 24H, C$H_3{}^{o}$-Mes). ^{13}C{^{1}H} (126 MHz, C_6D_6, 25 ºC) δ = 269.7 (t, ^1J(C,C) = 45 Hz, *C*O), 177.3 (t, ^1J(C,C) = 45 Hz, C^2), 141.1 ($C_6H_3{}^{o}$), 137.0 (Mes^{p}), 136.8 (Mes^{ipso}), 136.6 (Mes^{o}), 133.7 ($C_6H_3{}^{p}$), 132.2 ($C_6H_3{}^{ipso}$), 129.3 ($C_6H_3{}^{m}$), 128.9 (Mes^{m}), 21.3 (*C*$H_3{}^{p}$-Mes), 21.0 (*C*$H_3{}^{o}$-Mes). HRMS(ES−) *m/z*: calcd for ($C_{49}H_{53}O_3{}^{13}C_4$) [M+MeO]$^-$ 741.4134, found: 741.4118, (err [ppm] = 2.20). IR (Toluene): *v*/cm^{-1}: 1638 (^{13}C–O).

[(2,6-$Mes_2C_6H_3CO_2$)Fe(μ-$CO_2C_6H_3$-2,6-(2,6-Mes)$_2$]$_2$ (3^{Mes})

A solution of **1^{Mes}** (38.0 mg, 0.056 mmol) in C_6D_6 (0.6 mL) in a Young's tap NMR tube was exposed to an atmosphere of dry CO at room temperature whereupon an immediate colour change from yellow to red was observed. After 36 h a significant amount of crystalline material was formed which was determined by NMR and X-ray diffraction to be a mixture of **2^{Mes}** and **3^{Mes}** (11.3 mg). ^{1}H NMR (400 MHz, C_6D_6, 25 ºC): δ 62.3 (br s, $\Delta v_{1/2}$ = 523 Hz), 35.5

(s, $\Delta v_{1/2}$ = 168 Hz), 22.6 (s, $\Delta v_{1/2}$ = 159 Hz), 15.6 (br s, $\Delta v_{1/2}$ = 242 Hz), 14.3 (s, $\Delta v_{1/2}$ = 172 Hz), −3.9 (s, $\Delta v_{1/2}$ = 199 Hz), −6.6 (s, $\Delta v_{1/2}$ = 160 Hz), −8.4 (s, $\Delta v_{1/2}$ = 148 Hz), −10.7 (s, $\Delta v_{1/2}$ = 187 Hz), −14.9 (s, $\Delta v_{1/2}$ = 195 Hz), −24.2 (br s, $\Delta v_{1/2}$ = 286 Hz). IR (CH_2Cl_2): *v*/cm^{-1} 1610.

Reaction of (2,6-(2,6-Xyl)$_2$C$_6$H$_3$)$_2$Fe (1^{Xyl}) with CO

In a Young's flask, a stirred solution of **1^{Xyl}** (73.1 mg, 0.117 mmol) in toluene (10 mL) was exposed to an atmosphere of dry CO at room temperature whereupon an immediate colour change from yellow to red solution occurred. The reaction mixture was stirred for a further 6 days after which the solvent was removed *in vacuo* and the product extracted into toluene (5 mL), and pentane was carefully layered (5 mL) and kept at room temperature. This resulted in the precipitation of orange crystals suitable for X-ray diffraction revealing the presence of 1,3-[(2,6-(2,6-Xyl)$_2$C$_6$H$_3$]$_2$C$_4$O$_2$ (**2^{Xyl}**) and [(2,6-Xyl$_2$C$_6$H$_3$CO$_2$)Fe(μ-CO$_2$C$_6$H$_3$-2,6-(2,6-Xyl)$_2$]$_2$ (**3^{Xyl}**). The yields of **2^{Xyl}** and **3^{Xyl}** could not be accurately determined due their similar solubility which hindered their separation. Selected spectroscopic data of a mixture of **2^{Xyl}** and **3^{Xyl}** prepared *in situ*:

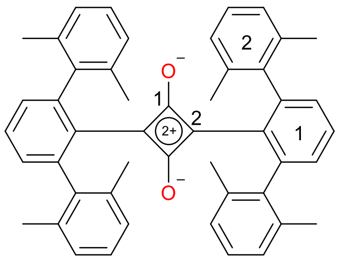

1,3-(2,6-Xyl$_2$C$_6$H$_3$)$_2$C$_4$O$_2$ (2^{Xyl}): ^{1}H NMR (400 MHz, C$_6$D$_6$, 25 °C): *δ* 6.95 (m, 2H, C$_6$H$_3^{p1}$), 6.88 (m, 8H, C$_6$H$_3^{m2}$), 6.73 (m, 4H, C$_6$H$_3^{p2}$), 6.55 (br d, 4H, C$_6$H$_3^{m1}$), 1.78 (s, 24H, C*H*$_3$-Xyl). ^{13}C{^{1}H} (101 MHz, C$_6$D$_6$, 25 °C): *δ* 260.8 (*C*^{1}O), 175.6 (C^2). HRMS(ES−) *m/z*: calcd. for C$_{48}$H$_{42}$O$_2$ [M]$^-$: 649.3112, found: 649.3097, (err [ppm] = 1.50). IR (Toluene): *v*/cm^{-1} 1675 (C–O). **[(2,6-Xyl$_2$C$_6$H$_3$CO$_2$)Fe(μ-CO$_2$C$_6$H$_3$-2,6-(2,6-Xyl)$_2$]$_2$ (3^{Xyl}):** ^{1}H NMR (400 MHz, C$_6$D$_6$, 25 °C): *δ* 54.8 (s, $\Delta v_{1/2}$ = 352 Hz), 30.7 (s, $\Delta v_{1/2}$ = 101 Hz), 26.4 (s, $\Delta v_{1/2}$ = 91 Hz), 23.4 (s, $\Delta v_{1/2}$ = 200 Hz), 21.5 (s, $\Delta v_{1/2}$ = 57 Hz), 16.0 (s, $\Delta v_{1/2}$ = 123 Hz), −3.6 (br, $\Delta v_{1/2}$ = 69 Hz), −9.8 (s, $\Delta v_{1/2}$ = 43 Hz), −16.9 (s, $\Delta v_{1/2}$ = 123 Hz), −22.12 (s, $\Delta v_{1/2}$ = 298 Hz), −30.7 (s, $\Delta v_{1/2}$ = 339 Hz). IR (CH_2Cl_2): *v*/cm^{-1} 1695.

(2,6-Naph$_2$C$_6$H$_3$)$_2$Fe(THF) (1^{Naph})

A solution of [Naph$_2$C$_6$H$_3$Li]$_2$ (200 mg, 0.297 mmol) in diethyl ether (25 mL) was added dropwise to a stirred suspension of FeCl$_2$(THF)$_{1.5}$ (56.0 mg, 0.238 mmol) in diethyl ether (25 mL) at −78 °C. The reaction was stirred at −78 °C for 1 h, then allowed to warm to −30 °C. The reaction was filtered at −30 °C, concentrated *in vacuo* at −30 °C, and the resulting orange/yellow solution transferred to a −30 °C freezer overnight. Orange crystals of (2,6-

$Naph_2C_6H_3)_2Fe(THF)$ (**1^{Naph}**) suitable for X-ray diffraction were obtained (116 mg, 27%). It should be noted that **1^{Naph}** is thermally sensitive and decomposes in solution at temperatures above −20 °C. ^1H NMR (400 MHz, C_6D_6, 25 ºC): δ 8.11 (d, br, ^3J(H,H) = 7.9 Hz, 1H), 7.71 (d, br, ^3J(H,H) = 8.0 Hz, 1H), 7.66 (d, br, ^3J(H,H) = 8.2 Hz, 1H), 7.56 (s, br, $\Delta v_{1/2}$ = 8.1 Hz), 7.43 (d, br, ^3J(H,H) = 8.8 Hz, 2H), 7.35 (s, br, $\Delta v_{1/2}$ = 14.0 Hz), 7.29 (s, br, $\Delta v_{1/2}$ = 24.0 Hz), 6.96 (s, br, $\Delta v_{1/2}$ = 10.9 Hz), 2.97 (s, br, $\Delta v_{1/2}$ = 27.7 Hz), 0.17 (s, br, $\Delta v_{1/2}$ = 12.0 Hz). μ_{eff} (Evans, C_6D_6, 25 ºC): 4.68 μ_B. Elemental analysis $C_{60}H_{54}FeO_2$: calc. C 83.51, H 6.31; found C 83.57, H 5.93. MS(EI) *m/z* = 658 (10%) $[(2,6\text{-}Naph_2C_6H_3)_2]^+$, 329 (50%) $[2,6\text{-}Naph_2C_6H_3]^+$, 202 (10%) $[2,6\text{-}Naph_2C_6H_3 - Naph]^+$. UV/vis ($Et_2O$): strong absorption below 450 nm with peak at λ_{max}/nm (ε/dm^3 mol^{-1} cm^{-3}) 1049 (28). IR (Nujol mull): v/cm^{-1} 1589 (wk), 1184 (wk, br), 1098 (wk, br), 1019 (wk), 798 (st), 777 (st), 723(st), 618 (wk), 568 (wk).

$(CO)_3Fe[C(2,6\text{-}Naph_2C_6H_3)OC(O)(2,6\text{-}Naph_2C_6H_3)]\cdot Et_2O$ (4)

A solution of $[Naph_2C_6H_3Li]_2$ (500 mg, 0.74 mmol) in diethyl ether (20 mL) was added dropwise to a stirred, cooled (−78 °C) suspension of $FeCl_2(THF)_{1.5}$ (175 mg, 0.74 mmol) in diethyl ether (20 mL). After stirring for 1 h at −78 °C, the reaction mixture was stored at −30 °C for 16 h. The reaction mixture was then filtered at −30 °C to afford a clear orange solution. This was exposed to an atmosphere of dry CO at −30 °C, with stirring, which resulted in an immediate colour change to dark orange/brown. The reaction was warmed to room temperature, with stirring, overnight and volatiles were removed *in vacuo* to afford a dark orange/brown solid.

The product decomposes slowly under atmospheric conditions, so the following step was performed in air. The crude product was purified by flash column chromatography (silica gel 60, eluting with 25% dichloromethane in hexane, R_f = 0.32) to afford the crude product as a bright red/orange solid. This was dried under high vacuum overnight, and recrystallised from (anhydrous, oxygen free) diethyl ether at −30 °C to afford pure $(CO)_3Fe[C(2,6\text{-}Naph_2C_6H_3)OC(O)(2,6\text{-}Naph_2C_6H_3)]\cdot Et_2O$ (**4**) as bright orange/red crystals (170 mg, 25%). Compound **4** displays complex NMR spectra at room temperature due to the presence of multiple conformers in solution, this is discussed in more detail in Supplementary Figs 8-11.

Elemental analysis: calc. for $C_{61}H_{44}FeO_6$: C 78.88, H 4.77; found 78.69, H 4.68. MS(EI) *m/z* = 858 (90%) $[M + 4H]^+$, 826 (1%) $[M – CO]^+$, 810 (3%) $[M – CO_2]^+$, 770 (2%) $[M – 3(CO)]^+$, 714 (4%) $[M – Fe(CO)_3]^+$. UV/vis (THF): strong absorption below 550 nm with peaks at λ_{max}/nm (ε/dm^3 mol^{-1} cm^{-3}) 744 (31), 920 (53), 1059 (19). IR (ATR): v/cm^{-1} 3055 (wk, C–H

stretch), 2967 (wk, C–H stretch), 2866 (wk, C–H stretch), 2043 (st, C≡O stretch), 1972 (st, C≡O stretch), 1954 (vs, C≡O stretch), 1593 (md, C=O stretch), 1568 (wk), 1506 (md), 1453 (wk), 1438 (wk), 1391 (md), 1323 (md), 1247 (md), 1203 (st), 971 (md), 919 (md), 907 (md), 798 (st), 774 (vs), 763 (st), 619 (st), 432 (md).

Reaction Monitoring *via* IR Spectroscopy

In a Young's flask, a stirred solution of $\mathbf{1}^{\mathbf{Mes}}$ (72.2 mg, 0.106 mmol) or $\mathbf{1}^{\mathbf{Xyl}}$ (73.1 mg, 0.117 mmol) in toluene (8 mL) was exposed to an atmosphere of dry CO gas whereupon an immediate colour change from yellow to red was observed. Aliquots of the reaction solution (0.4 mL) were removed from the reaction vessel periodically under an atmosphere of CO and transferred to a separate Young's flask, after which the solvent was removed and the resulting red solid was dried for *ca.* 1 h. The sample was dissolved in toluene (0.4 mL) under an argon atmosphere and transferred to a pre-purged solution IR cell and the IR spectrum was recorded (see Supplementary Fig. 17).

Reaction Monitoring *via* NMR Spectroscopy

Reaction of 1^{Mes}

In a Young's NMR tube $\mathbf{1}^{Mes}$ (38.0 mg, 0.056 mmol) was dissolved in C_6D_6 (0.6 mL) with cumene (16 μL, 0.115 mmol) as an internal standard, and an initial ^1H NMR spectra was measured. The sample was then exposed to an atmosphere of dry CO gas whereupon an immediate colour change from yellow to red was observed, and the reaction was monitored by ^1H NMR spectroscopy. Conversion was quantified by integration of ^1H NMR spectra: 92% of $\mathbf{2}^{Mes}$ (3 days at room temperature).

Reaction of 1^{Xyl}

In a Young's NMR tube $\mathbf{1}^{Xyl}$ (38.0 mg, 0.061 mmol) was dissolved in C_6D_6 (0.6 mL) with cumene (16 μL, 0.115 mmol) as an internal standard, and an initial ^1H NMR spectra was measured. The sample was then exposed to an atmosphere of dry CO gas whereupon an immediate colour change from yellow to red was observed, and the reaction was monitored by ^1H NMR spectroscopy for 6 days.

Precise determination of conversion by integration was not possible in this experiment, due to the limited solubility of the products ($\mathbf{2}^{Xyl}$/$\mathbf{3}^{Xyl}$) which precipitated during the reaction. However, there was no evidence of byproduct formation, and analysis of both the solution and precipitated solid revealed $\mathbf{2}^{Xyl}$ and $\mathbf{3}^{Xyl}$ to be the only terphenyl-containing compounds.

Further Reaction of Compound 4 with CO

A solution of compound **4** (*ca.* 5 mg) in C_6D_6 was prepared in a Young's NMR tube under an inert atmosphere. The solvent was degassed by three freeze-pump-thaw cycles, and the sample exposed to an atmosphere of CO gas. The sample was monitored by ^1H and ^{13}C NMR spectroscopy after 5 h at room temperature, revealing the presence of dissolved CO gas (δ_C 184.5) but no reaction of compound **4**. The sample was then heated to 80 °C, with NMR spectra recorded after 8h and 14h. No appreciable further reaction was observed by NMR.

EPR Spectroscopy

In a typical experiment, to a Young's tap modified quartz EPR tube containing **2^{Mes}**, **2^{Mes}-^{13}C** or **2^{Xyl}** (*ca.* 0.01 mmol) in CH_2Cl_2 (*ca.* 0.4 mL) was added excess Cp_2Co (*ca.* 0.02 mmol), to generate the corresponding radical anion (**$2^{Mes\bullet-}$**, **$2^{Mes\bullet-}$-^{13}C** or **$2^{Xyl\bullet-}$** respectively) *in situ*. An EPR spectrum was then recorded at room temperature. Parameters for simulated and experimental spectra are given in Supplementary Table 2.

Crystallographic Methods

Under a flow of nitrogen, crystals suitable for analysis by X-ray diffraction were quickly removed from the crystallisation vessel and covered in YR-1800 perfluoropolyether oil. Crystals were mounted on a MiTeGen MicroMount™ and cooled rapidly in a cold stream of nitrogen using an Oxford Cryostreams open flow cryostat.[4] Single crystal X-ray diffraction data were collected on an Agilent SuperNova diffractometer (mirror-monochromated Cu-Kα radiation source; λ = 1.54184 Å; ω scans), equipped with either an Atlas, AtlasS2 or TitanS2 detector. Cell parameters were refined from the observed positions of all strong reflections in each data set and absorption corrections were applied using a Gaussian numerical method with beam profile correction (CrysAlisPro).[5] The structures were solved either by direct or iterative methods and all non-hydrogen atoms refined by full-matrix least-squares on all unique F^2 values with anisotropic displacement parameters. Hydrogen atoms were refined with constrained geometries and riding thermal parameters. Programs used include CrysAlisPro[5] (control of Supernova, data integration and absorption correction), SHELXL[6] (structure refinement), SHELXS[7] (structure solution), SHELXT[8] (structure solution), OLEX2[9] (molecular graphics). CIF files were checked using checkCIF[10] by Dr William Lewis and Prof. Alexander Blake at the University of Nottingham Crystal Structure Service. CCDC-1589889-1589895 contains the supplementary data for these compounds. These data can be obtained free of charge from The Cambridge Crystallographic Data Centre *via* www.ccdc.cam.ac.uk/data_request/cif.

Crystal data for (2,6-(2,6-Xyl)$_2$C$_6$H$_3$)$_2$Fe (1^{Xyl})

$C_{44}H_{42}Fe$ (M = 626.62 g/mol): tetragonal, space group P4$_3$ (no. 78), a = 10.48569(11) Å, c = 30.7962(6) Å, V = 3386.03(9) Å^3, Z = 4, T = 120(2) K, μ(Cu$K\alpha$) = 3.778 mm^{-1}, D_{calc} = 1.229 g/cm^3, 21453 reflections measured (8.432° ≤ 2Θ ≤ 148.558°), 6709 unique (R_{int} = 0.0422, R_{sigma} = 0.0341) which were used in all calculations. The final R_1 was 0.0308 (I > 2σ(I)) and wR_2 was 0.0794 (all data).

Crystal data for 1,3-(2,6-Mes$_2$C$_6$H$_3$)$_2$C$_4$O$_2$ (2^{Mes})

$C_{55}H_{57}O_2$ (M = 750.00 g/mol): triclinic, space group P-1 (no. 2), a = 11.0869(8) Å, b = 11.7248(6) Å, c = 17.6257(11) Å, α = 79.831(5)°, β = 79.293(6)°, γ = 83.642(5)°, V = 2208.7(3) Å^3, Z = 2, T = 120.00(14) K, μ(Cu$K\alpha$) = 0.506 mm^{-1},D_{calc} = 1.128 g/cm^3, 18751 reflections measured (7.686° ≤ 2Θ ≤ 147.408°), 8637 unique (R_{int} = 0.0692, R_{sigma} = 0.0934)

which were used in all calculations. The final R_1 was 0.0655 (I > 2σ(I)) and wR_2 was 0.1813 (all data).

Crystal data for 1,3-(2,6-Xyl$_2$C$_6$H$_3$)$_2$C$_4$O$_2$ (2^{Xyl})

$C_{24}H_{21}O$ (M = 325.41 g/mol): orthorhombic, space group *Ccce* (no. 68), a = 11.7258(5) Å, b = 19.0058(8) Å, c = 16.1304(7) Å, V = 3594.8(3) Å^3, Z = 8, T = 120(2) K, μ(Cu$K\alpha$) = 0.551 mm^{-1}, D_{calc} = 1.203 g/cm^3, 4697 reflections measured (9.306° ≤ 2Θ ≤ 147.156°), 1770 unique (R_{int} = 0.0317, R_{sigma} = 0.0295) which were used in all calculations. The final R_1 was 0.0410 (I > 2σ(I)) and wR_2 was 0.1114 (all data).

Crystal data for [(2,6-Mes$_2$C$_6$H$_3$CO$_2$)Fe(μ-CO$_2$C$_6$H$_3$-2,6-(2,6-Mes)$_2$]$_2$ (3^{Mes})

$C_{106}H_{100}D_6Fe_2O_8$ (M = 1625.64 g/mol): triclinic, space group *P*-1 (no. 2), a = 12.5782(6) Å, b = 13.1227(5) Å, c = 14.1774(7) Å, α = 67.310(4)°, β = 83.233(4)°, γ = 85.506(3)°, V = 2142.59(18) Å^3, Z = 1, T = 120(2) K, μ(Cu$K\alpha$) = 3.175 mm^{-1}, D_{calc} = 1.260 g/cm^3, 31251 reflections measured (6.788° ≤ 2Θ ≤ 147.506°), 8481 unique (R_{int} = 0.0252, R_{sigma} = 0.0186) which were used in all calculations. The final R_1 was 0.0383 (I > 2σ(I)) and wR_2 was 0.1124 (all data).

Crystal data for [(2,6-Xyl$_2$C$_6$H$_3$CO$_2$)Fe(μ-CO$_2$C$_6$H$_3$-2,6-(2,6-Xyl)$_2$]$_2$ (3^{Xyl})

$C_{92}H_{84}Fe_2O_8$ (M = 1429.29 g/mol): monoclinic, space group *I*2/*a* (no. 15), a = 22.1583(5) Å, b = 11.7559(3) Å, c = 32.9915(8) Å, β = 91.687(2)°, V = 8590.3(4) Å^3, Z = 4, T = 120(2) K, μ(Cu$K\alpha$) = 3.109 mm^{-1}, D_{calc} = 1.105 g/cm^3, 19142 reflections measured (7.984° ≤ 2Θ ≤ 148.418°), 8552 unique (R_{int} = 0.0310, R_{sigma} = 0.0351) which were used in all calculations. The final R_1 was 0.0838 (I > 2σ(I)) and wR_2 was 0.2615 (all data).

Crystal data for (2,6-Naph$_2$C$_6$H$_3$)$_2$Fe(THF) (1^{Naph})

$C_{60}H_{52}FeO_2$ (M = 860.86 g/mol): monoclinic, space group $P2_1/c$ (no. 14), a = 16.976(7) Å, b = 17.131(7) Å, c = 15.512(6) Å, β = 97.096(9)°, V = 4477(3) Å^3, Z = 4, T = 90(2) K, μ(Mo$K\alpha$) = 0.382 mm^{-1}, D_{calc} = 1.277 g/cm^3, 22421 reflections measured (4.114° ≤ 2Θ ≤ 50°), 7856 unique (R_{int} = 0.1101, R_{sigma} = 0.1394) which were used in all calculations. The final R_1 was 0.0868 (I > 2σ(I)) and wR_2 was 0.1890 (all data).

Crystal data for (CO)$_3$Fe[C(2,6-Naph$_2$C$_6$H$_3$)OC(O)(2,6-Naph$_2$C$_6$H$_3$)] (4)

$C_{61}H_{44}O_6Fe$ (M = 928.81 g/mol): triclinic, space group *P*-1 (no. 2), a = 10.4451(6) Å, b = 11.4520(5) Å, c = 20.3946(10) Å, α = 103.562(4)°, β = 100.736(4)°, γ = 95.107(4)°, V = 2307.4(2) Å^3, Z = 2, T = 90(2) K, μ(Cu$K\alpha$) = 3.063 mm^{-1}, D_{calc} = 1.337 g/cm^3, 17055

reflections measured (8.02° ≤ 2Θ ≤ 133.184°), 8159 unique (R_{int} = 0.0381, R_{sigma} = 0.0471) which were used in all calculations. The final R_1 was 0.0471 (I > 2σ(I)) and wR_2 was 0.1165 (all data).

Computational Methods

Computational Analysis of Squaraine

Initial calculations were performed on a model of **2^Mes^** and **2^Xyl^** (**2a**) in which in which the flanking mesityl and xylyl substituents were replaced by a phenyl group. Density functional theory (DFT), using the ωB97X-D functional[12] and 6-31G(d) basis set,[13] was used to optimize the geometry. Further single-point energy calculations were performed on a reduced model **2b** in which the terphenyl substituents were replaced with phenyl groups (Supplementary Fig. 20). These calculations were carried out using the restricted active space self-consistent field (RASSCF) approach, with an active space of 18 electrons in 18 orbitals, with a configuration interaction restricted to single, double and triple excitations (SDT); the RAS2 space was not used. The orbitals correspond to the six π and six π* orbitals of the phenyl rings, the two C=O π and two C=O π* orbitals, and two further orbitals located on the bridging carbon atoms which were strongly interacting with the π orbitals at the non-planar geometry from the DFT calculations. The RASSCF calculations[14] indicate that a closed-shell singlet is the predominant electronic configuration, even when the singlet calculation is performed starting with the RASSCF optimized triplet orbitals. Geometry optimized co-ordinates for **2a** and **2b** are given in Supplementary Tables 5 and 6 respectively.

Further DFT calculations were performed on the neutral and anionic forms of **2a**, using the ωB97X-D functional and 6-31G(d) basis set. Frequency calculations were performed with all carbon atoms having a mass of 12.011 g mol^{-1}, and a second set of frequency calculations in which the four carbon atoms of the bridging square unit had a mass of 13.00336 g mol^{-1}**.** All frequencies were scaled by 0.95, to account approximately for anharmonic effects. The scaled frequencies are given in Supplementary Table 3. All calculations were performed with the Q-Chem software package.[15]

Calculation of hyperfine coupling constants

Single point calculations using the gas phase geometry optimized structure of **$2a^{\bullet-}$** were used to calculate hyperfine coupling constants for the ^{13}C and ^{1}H nuclei of **$2a^{\bullet-}$**. The calculations employed the PBE0 functional[16] and the EPR-II basis,[17] and were carried out using the ORCA software package.[18] Calculated hyperfine coupling constants are given in

Supplementary Table 4. Geometry optimized co-ordinates for **2a**$^{\bullet-}$ are given in Supplementary Table 7.

Computational Analysis of Proposed Reaction Pathway

Geometry optimisations were performed for **D**$^{\mathbf{Naph}}$, **E**$^{\mathbf{Naph}}$, **D**$^{\mathbf{Xyl}}$, **E**$^{\mathbf{Xyl}}$ (Supplementary Fig. 22) and the transition state between each using density functional theory (DFT), with the ωB97X-D functional[12] and LANL2DZ basis set and effective core potential.[19,20] The empirical dispersion corrections afforded by this method were necessary to correctly describe the geometrical parameters. Minima and transition states were confirmed using vibrational frequency calculations. Energies were refined using the B3LYP functional[21] and Stuttgart-Bonn basis set / ECP[22,23] (denoted SRSC within Q-Chem), incorporating an effective core potential for the Fe atom, and 6-311G(d) for all other electrons. The polarisable continuum model (PCM) was used, with a dielectric constant of 2.27, representing benzene.[24-26] Geometry optimised co-ordinates for **D**$^{\mathbf{Naph}}$, **E**$^{\mathbf{Naph}}$, **D**$^{\mathbf{Xyl}}$, **E**$^{\mathbf{Xyl}}$ and the transition state between each are given in Supplementary Tables 8–13.

Supplementary Discussion

NMR spectra of Compound 4

Compound **4** displays complex ^{1}H and ^{13}C{^{1}H} NMR spectra at room temperature, which is attributed to the presence of multiple conformers in solution which do not interconvert at room temperature. This is a consequence of the naphthyl flanking groups, which can adopt *syn-* or *anti*-conformations (Supplementary Fig. 8).[11]

The ^{1}H and ^{13}C{^{1}H} NMR spectra of compound **4** (298 K, C_6D_6) are shown in Supplementary Fig. 9 and Supplementary Fig. 10. Of note is the ^{13}C{^{1}H} NMR spectrum (Supplementary Fig. 10) where clusters of 3 signals are observed at δ_C = 211 and 173 ppm. These are attributed to the carbene and carbonyl carbons respectively and indicate the presence of three conformers in solution (*syn*/*syn*, *syn*/*anti*, and *anti*/*anti* arrangements of the naphthyl flanking groups).

VT-NMR was carried out on a solution of **4** in 1,1,2,2-tetrachloroethane-d_2. However, the compound underwent decomposition at 363 K, before reaching the coalescence point. Some differences were observed in the spectra acquired at 298 K and 343 K, although not enough to provide conclusive evidence for the interconversion of conformers (Supplementary Fig. 11).

Supplementary Figures

Crystal Structure Figures

Supplementary Figure 1: Crystal Structure of 1^{Xyl}

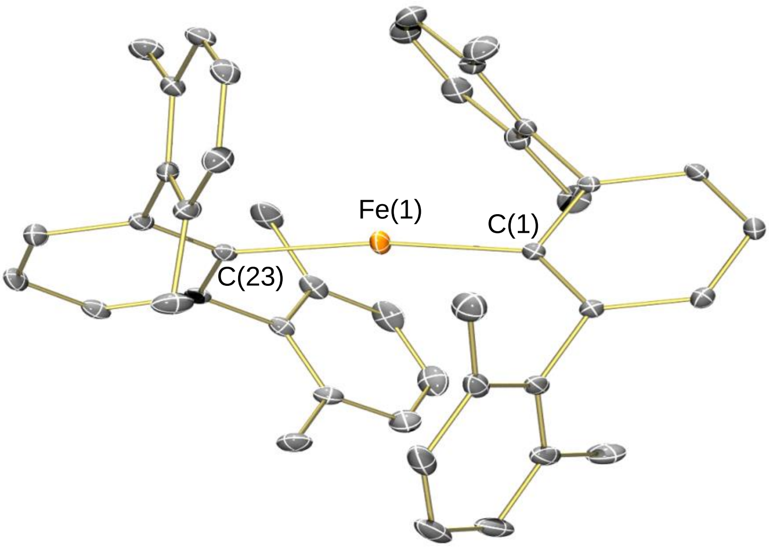

Molecular structure of **1**^{Xyl} with anisotropic thermal parameters set at 50% probability. Hydrogen atoms have been omitted for clarity.

Supplementary Figure 2: Crystal Structure of 2^{Xyl}

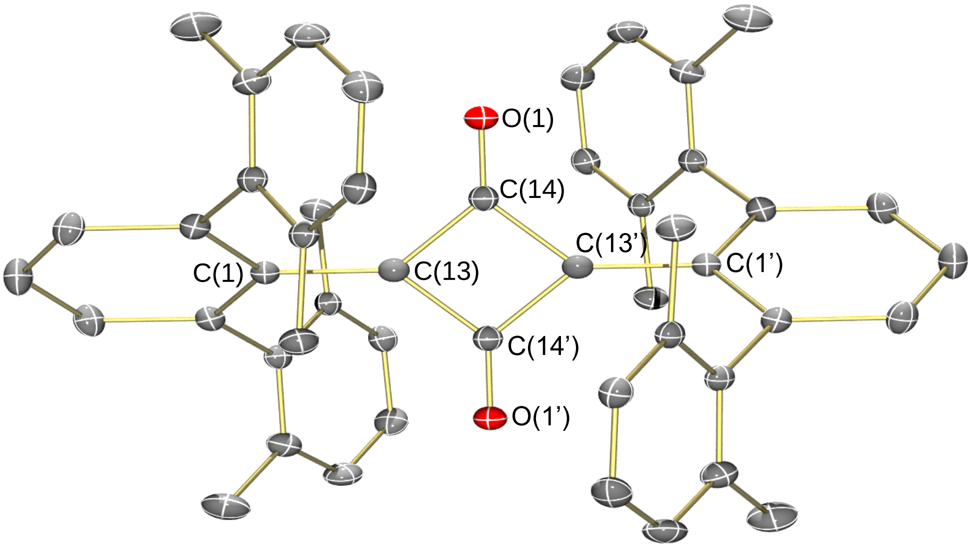

Molecular structure of **2**^{Xyl} with anisotropic thermal parameters set at 50% probability. Hydrogen atoms have been omitted for clarity. Selected bond distances (Å) and angles (°) for **2**^{Xyl}: O(1)–C(14) 1.212(2), C(13)–C(14) 1.4837(18), C(1)plane–C_4O_2 50.93(5).

Supplementary Figure 3: Crystal Structure of 3^{Xyl}

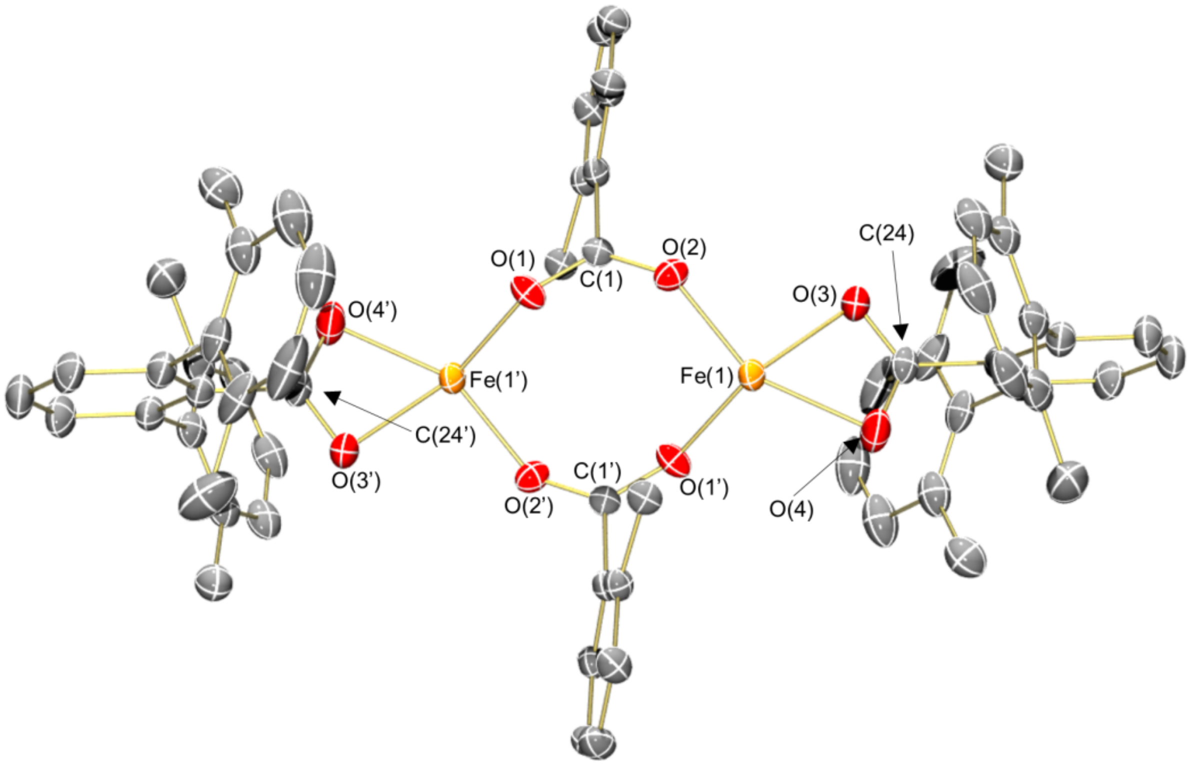

Molecular structure of $\mathbf{3}^{Xyl}$ with anisotropic thermal parameters set at 50% probability. Hydrogen atoms have been omitted for clarity. Selected bond distances (Å) and angles (°) for $\mathbf{3}^{Xyl}$: Fe(1)–O(1) 1.978(2), Fe(1)–O(2) 1.961(2), Fe(1)–O(3) 2.082(3), Fe(1)–O(4) 2.149(3), O1–Fe(1)–O(2) 98.39(10), O(3)–Fe(1)–O(4) 61.55(10).

Supplementary Figure 4: Crystal Structure of 1^{Naph}

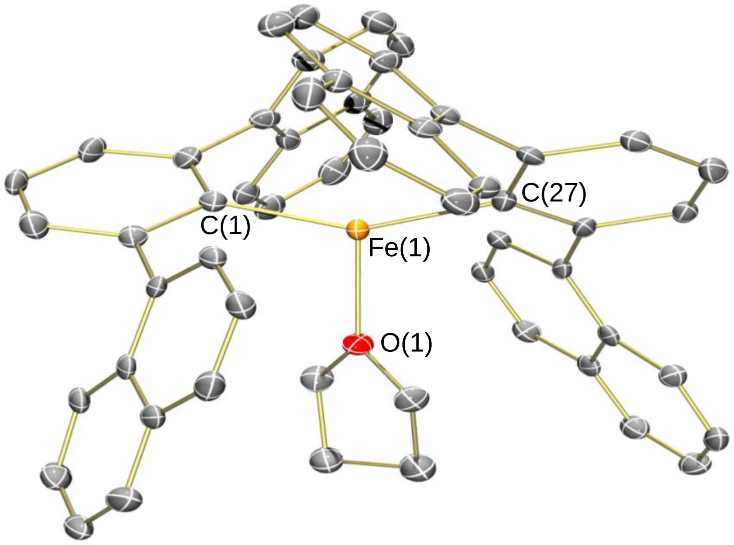

Molecular structure of **1^{Naph}** with anisotropic thermal parameters set at 50% probability. Hydrogen atoms have been omitted for clarity.

NMR Spectra and Related Figures

Supplementary Figure 5: Selected NMR Spectra of 2^{Mes}

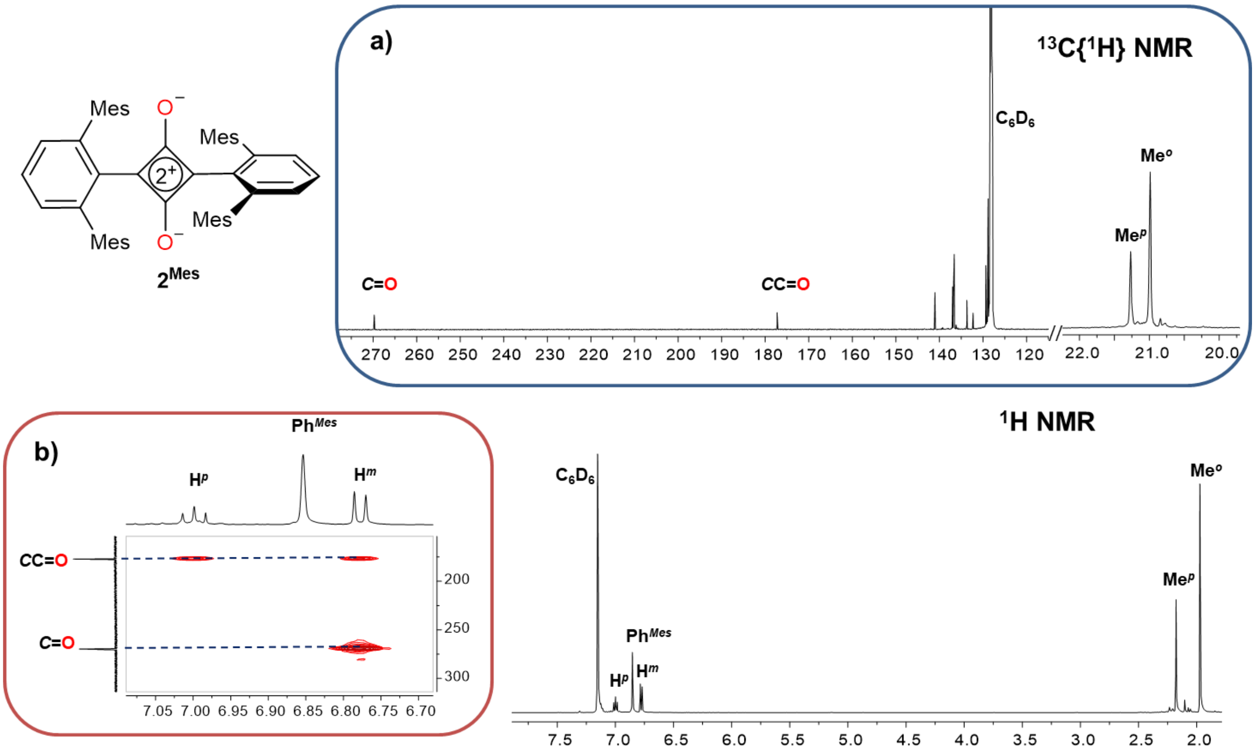

Selected region of the ^{1}H, $^{13}C\{^{1}H\}$ (framed in blue) and $^{1}H,^{13}C$-HMBC (framed in red) NMR spectra of squaraine 1,3-(2,6-$Mes_2C_6H_3)_2C_4O_2$ (**2^{Mes}**) in C_6D_6.

Supplementary Figure 6: $^{13}C,^{13}C$ COSY NMR Spectra of 2^{Mes}-^{13}C

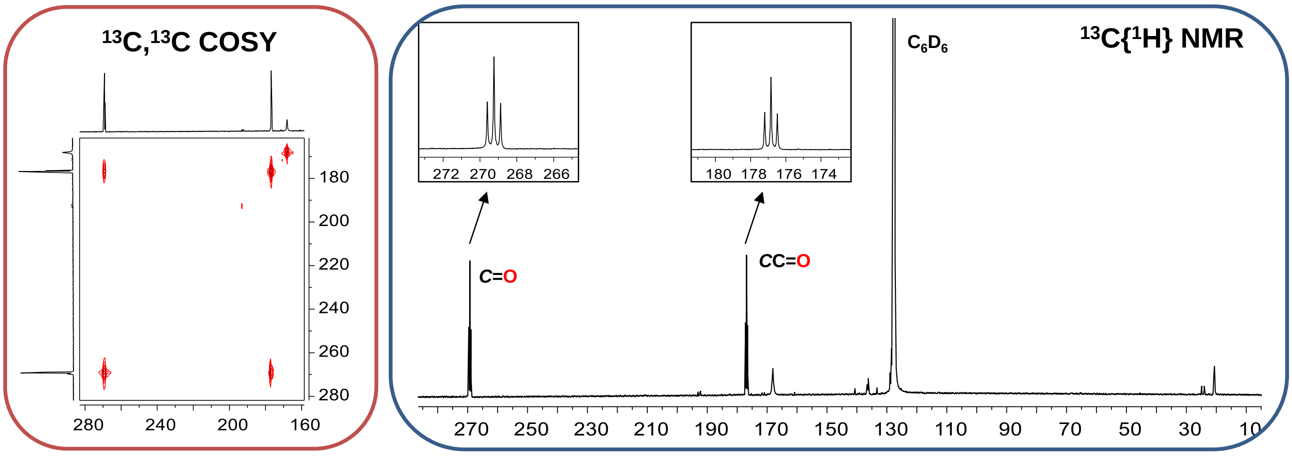

Selected region of the $^{13}C\{^{1}H\}$ (framed in blue) and $^{13}C,^{13}C$-COSY (framed in red) NMR spectra of squaraine 1,3-(2,6-$Mes_2C_6H_3)_2{}^{13}C_4O_2$ (**2^{Mes}-^{13}C**) in C_6D_6.

Supplementary Figure 7: Selected region of $^{13}C\{^1H\}$ NMR Spectra of 2^{Mes}-^{13}C

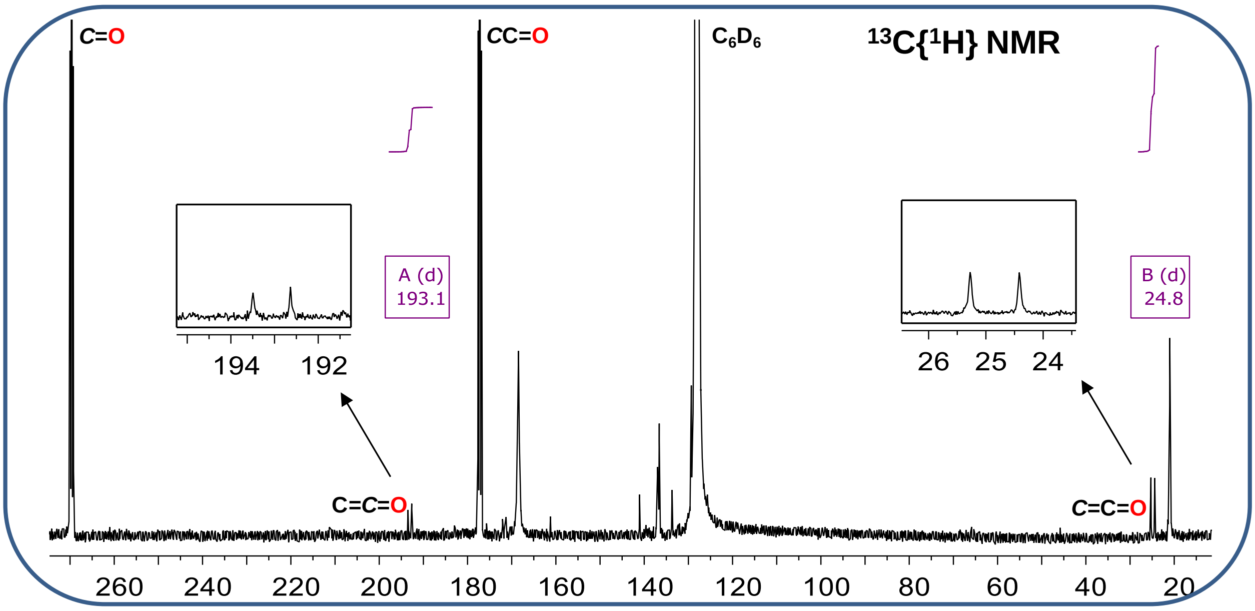

Selected region of the $^{13}C\{^1H\}$ (framed in blue) NMR spectra of squaraine 1,3-(2,6-$Mes_2C_6H_3)_2{}^{13}C_4O_2$ (**2^{Mes}-^{13}C**) with small amount of ketenyl-type intermediate at 193.6 (d, ^1J(C,C) = 108 Hz, C=*C*=O) and 24.8 (d, ^1J(C,C) = 108 Hz, *C*=C=O)].

Supplementary Figure 8: Structure of compound 4 and possible *syn-* and *anti-* conformers

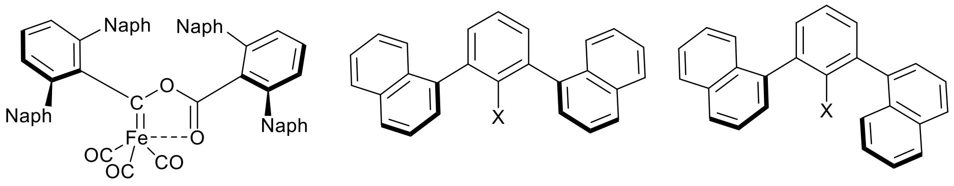

4 *syn* *anti*

Structure of compound **4** (left) and possible *syn-* (centre) and *anti-* (right) conformations arising from the presence of naphthyl flanking groups (Naph = 1-$C_{10}H_7$). These conformers do not readily interconvert, and result in complex ^{1}H and ^{13}C NMR spectra (see Supplementary Discussion).

Supplementary Figure 9: ^{1}H NMR Spectrum of 4 in C_6D_6

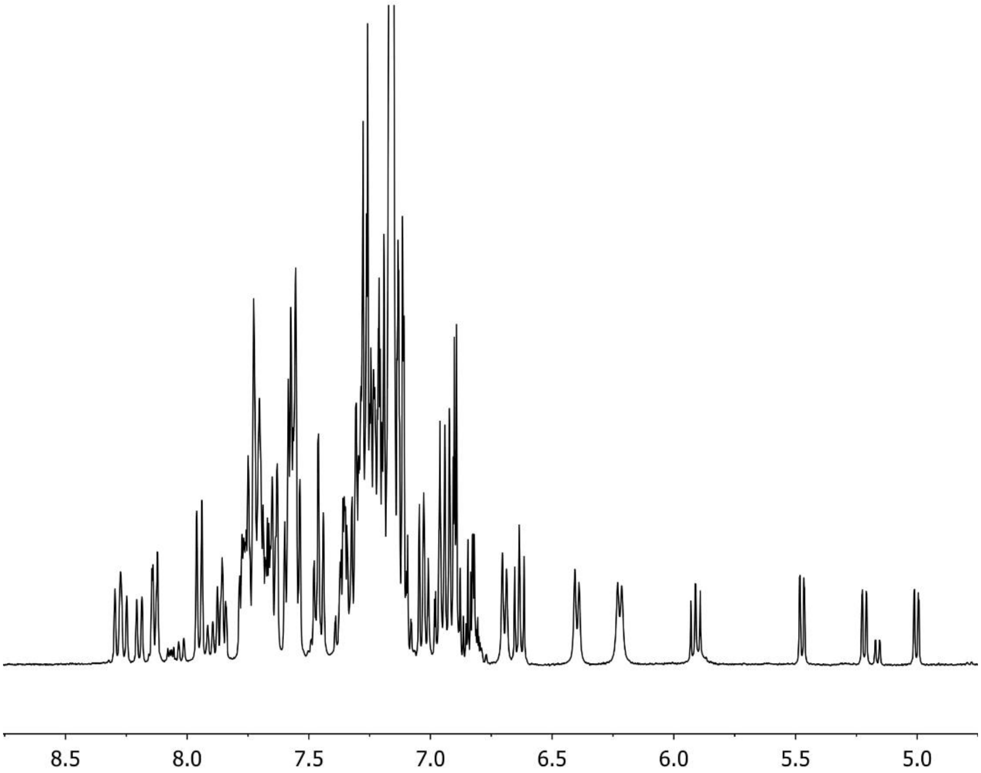

Selected region of the ^{1}H NMR spectrum of compound **4** (C_6D_6, 298 K, signals referenced to solvent residual peak).

Supplementary Figure 10: ^{13}C NMR Spectrum of 4 in C_6D_6

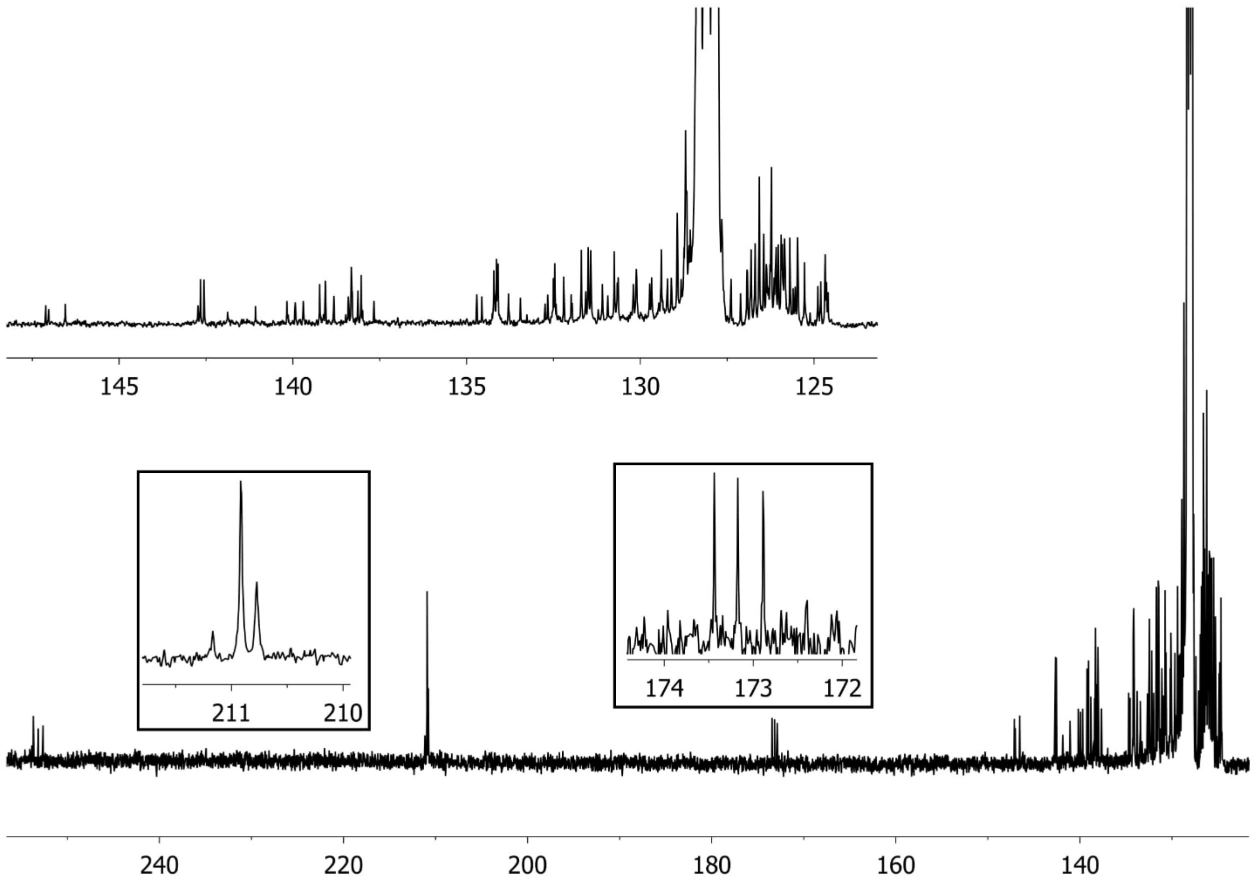

Selected regions of the $^{13}C\{^1H\}$ NMR spectrum of compound **4** (C_6D_6, 298 K, signals referenced to solvent residual peak).

Supplementary Figure 11: Variable Temperature ^{1}H NMR spectra of 4

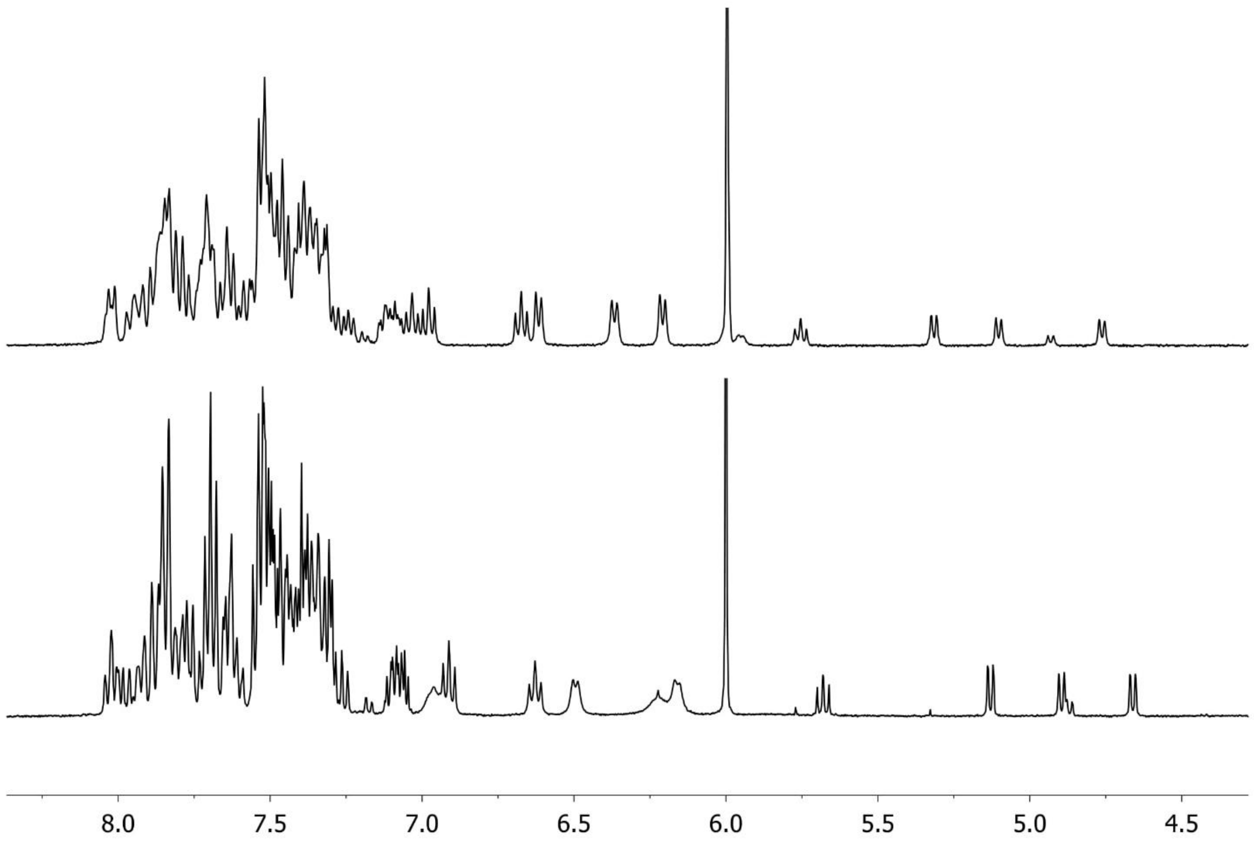

Selected region of the ^{1}H NMR spectrum of compound **4** in $(CDCl_2)_2$ at 298 K (bottom) and 343 K (top). Signals referenced to solvent residual peak.

IR and EPR Spectroscopy Figures

Supplementary Figure 12: Solution IR Spectra of 2^{Mes}

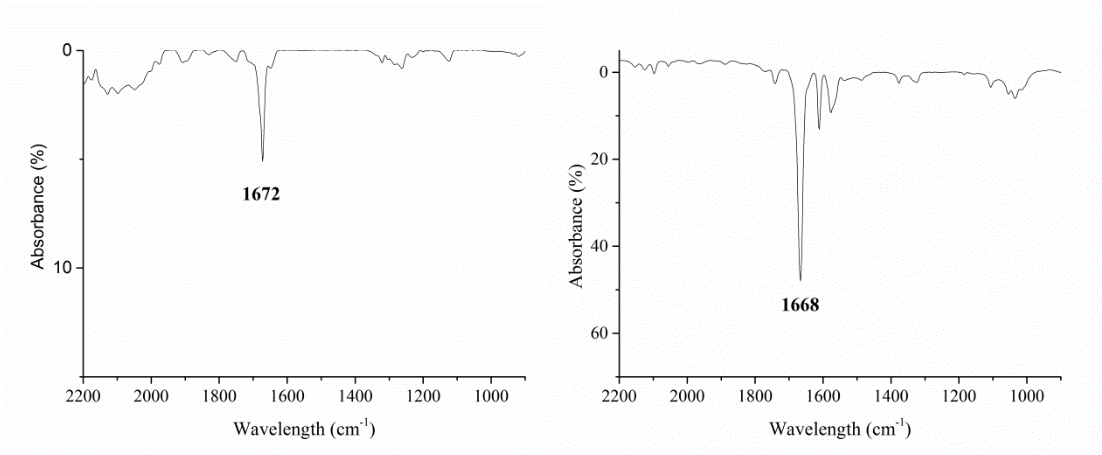

IR spectra of **2^{Mes}** in toluene (left) and CH_2Cl_2 (right).

Supplementary Figure 13: Cyclic Voltammograms of 2^{Mes} (Reduction)

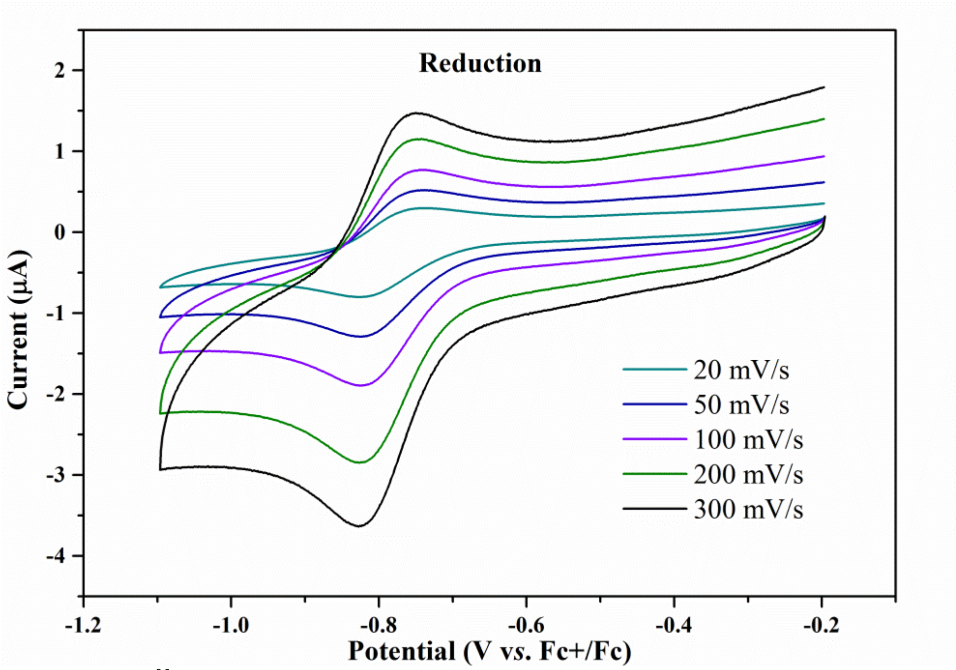

Cyclic voltammograms of **2^{Mes}** in CH_2Cl_2 containing 0.4 M [$^{n}Bu_4N$][BF_4] as the supporting electrolyte.

Supplementary Figure 14: Cyclic Voltammograms of 2^{Mes} (Oxidation)

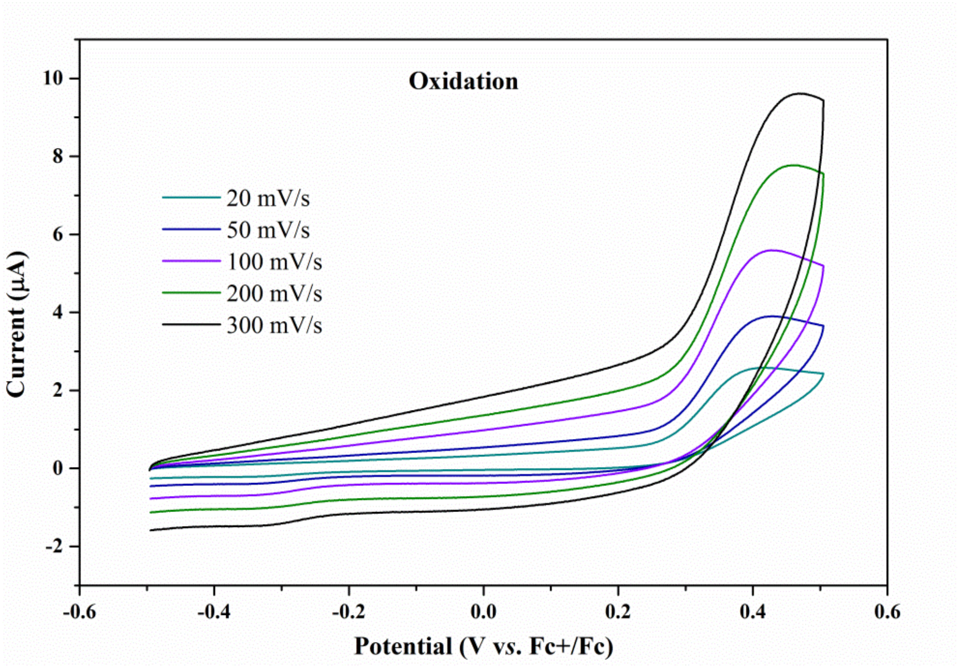

Cyclic voltammograms of **2^{Mes}** in CH_2Cl_2 containing 0.4 M [^{n}Bu$_4$N][BF$_4$] as the supporting electrolyte.

Supplementary Figure 15: IR Spectra from the reaction of 1^{Mes} with CO (toluene)

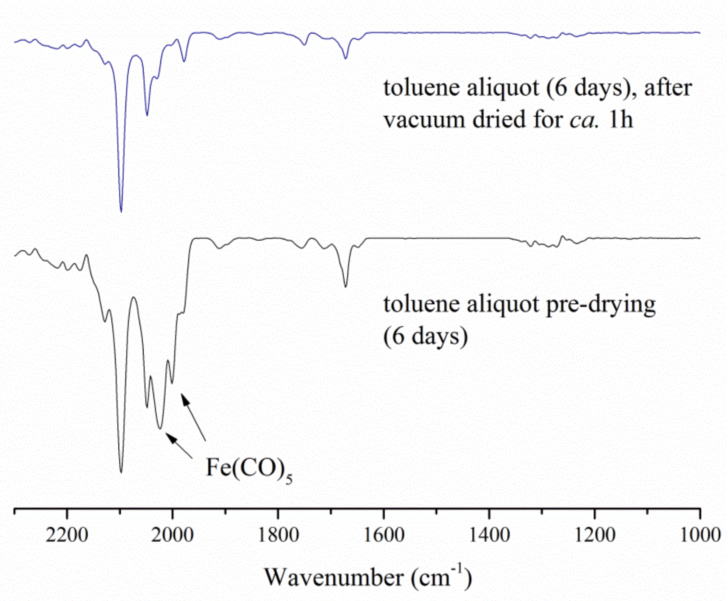

IR spectra in toluene at room temperature obtained from reaction of **1^{Mes}** with CO after 6 days, before and after vacuum drying to remove $Fe(CO)_5$.

Supplementary Figure 16: IR Spectra from the reaction of 1^{Mes} with CO (CH_2Cl_2)

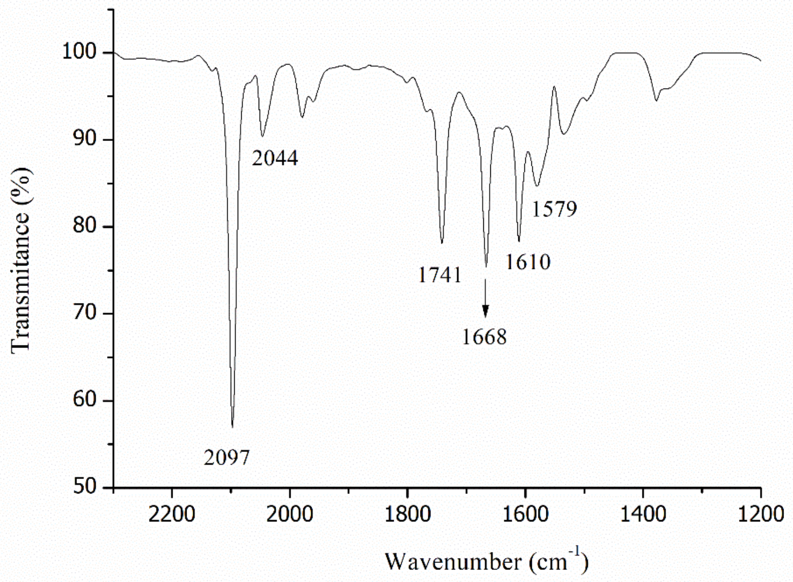

IR spectra in CH_2Cl_2 at room temperature obtained from reaction of 1^{Mes} with CO, after 6 days of reaction in toluene, removal of toluene by *in vacuo* and dissolving in CH_2Cl_2.

Supplementary Figure 17: IR Spectra from the reaction of 1^{Xyl} with CO (toluene)

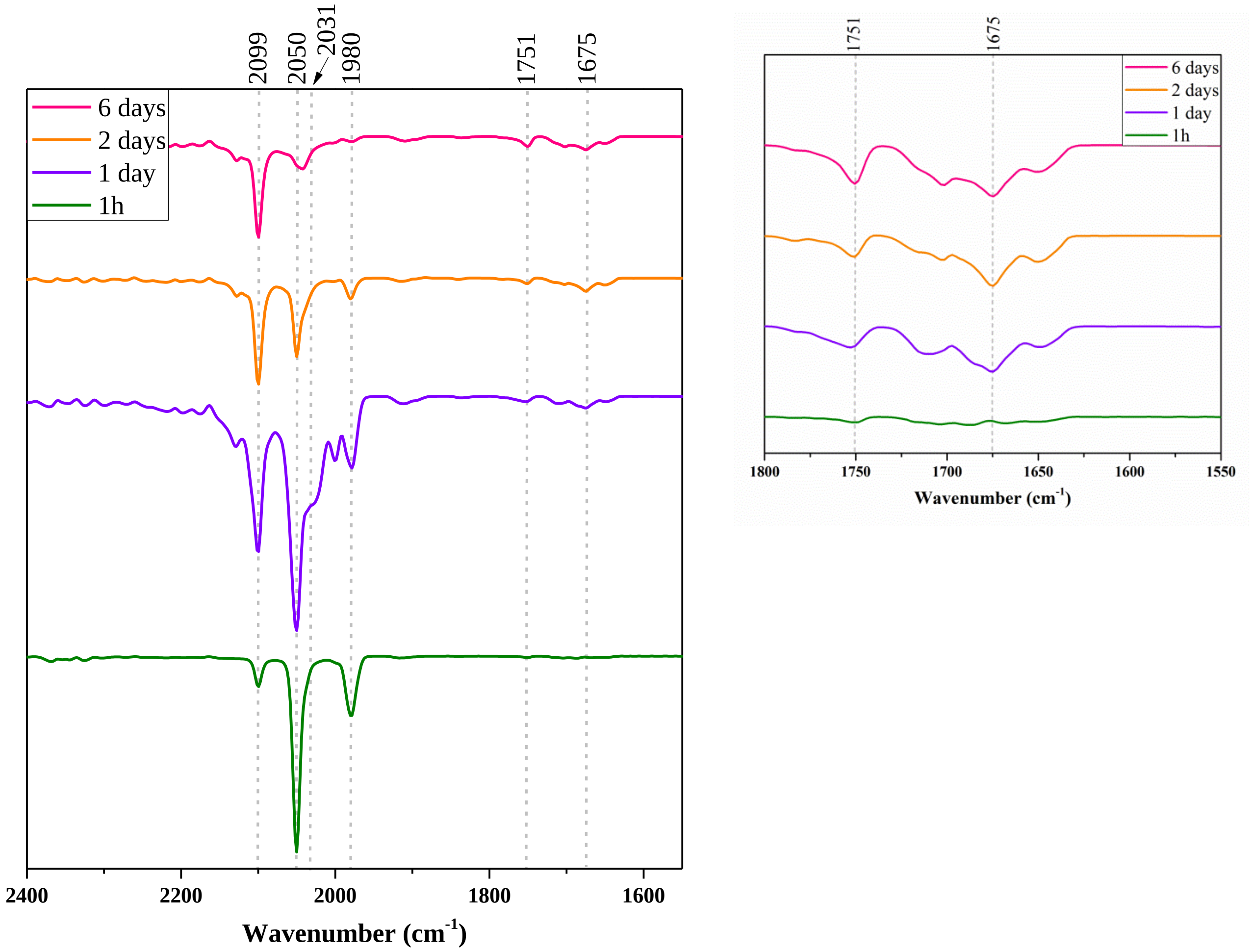

IR spectra in toluene at room temperature obtained from reaction of **1**^{Xyl} with CO.

Supplementary Figure 18: IR Spectra from the reaction of 1^{Xyl} with CO (CH_2Cl_2)

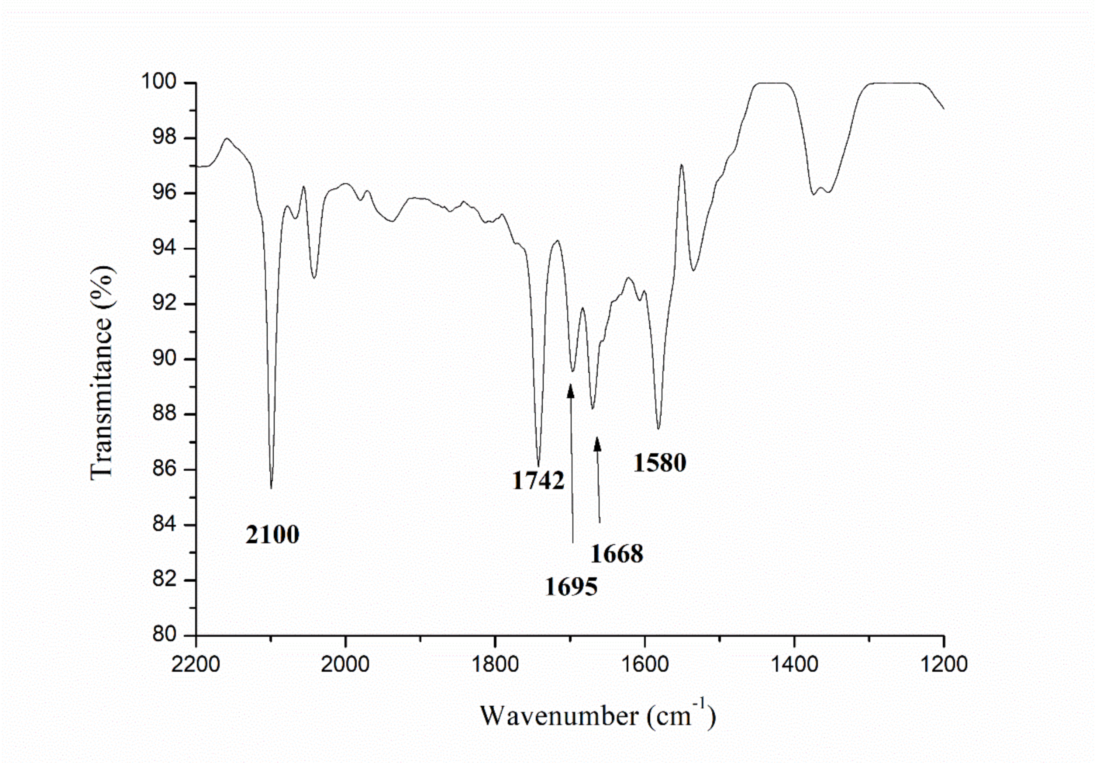

IR spectra in CH_2Cl_2 at room temperature obtained from reaction of **1^{Xyl}** with CO, after 3 days of reaction in toluene, removal of toluene by vacuum drying and dissolving in CH_2Cl_2.

Supplementary Figure 19: EPR Spectrum of $2^{Xyl\bullet-}$

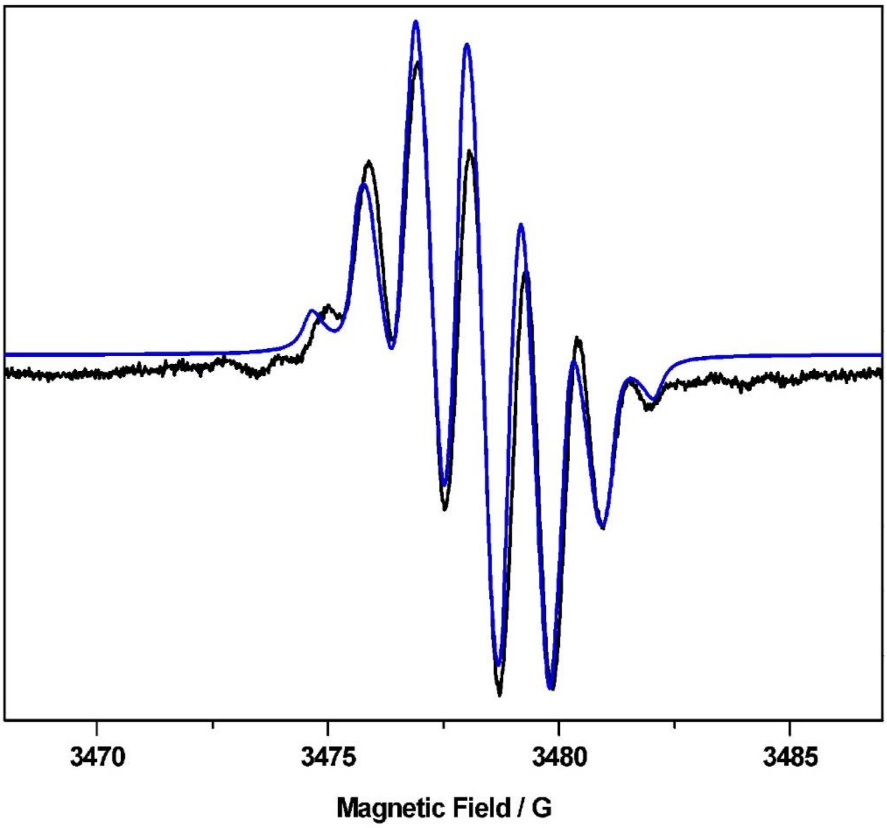

Experimental X-band EPR spectrum of $\mathbf{2}^{Xyl\bullet-}$ (black trace) recorded as a fluid solution in CH_2Cl_2 at room temperature. The simulated spectrum is given in blue and parameters used for the simulation are listed in Table S2. Coupling to natural abundance ^{13}C was noted but not sufficient resolved to be included in the simulation parameters.

Computational Figures

Supplementary Figure 20: Structure of 2b

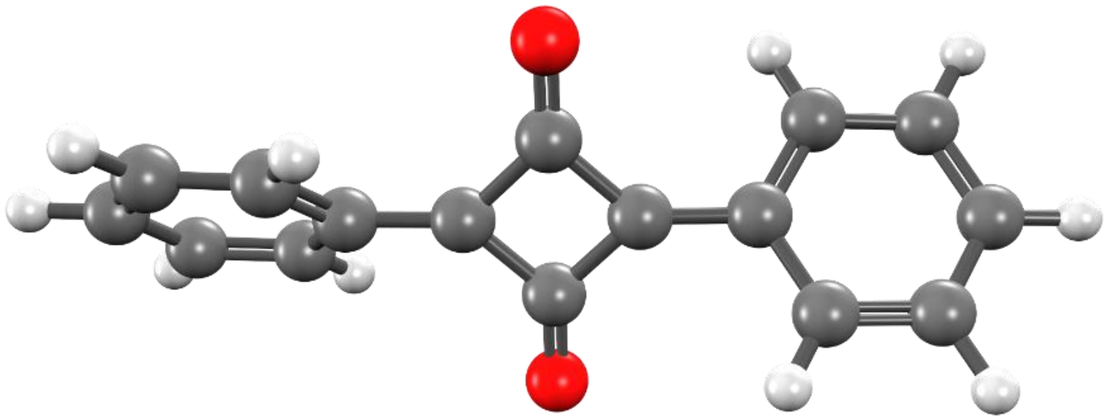

Molecular structure of the reduced model compound of $\mathbf{2}^{\mathbf{Mes}}$ and $\mathbf{2}^{\mathbf{Xyl}}$ (**2b**) used in the RASSCF calculations.

Supplementary Figure 21: Computed structure and spin density for 2a•−

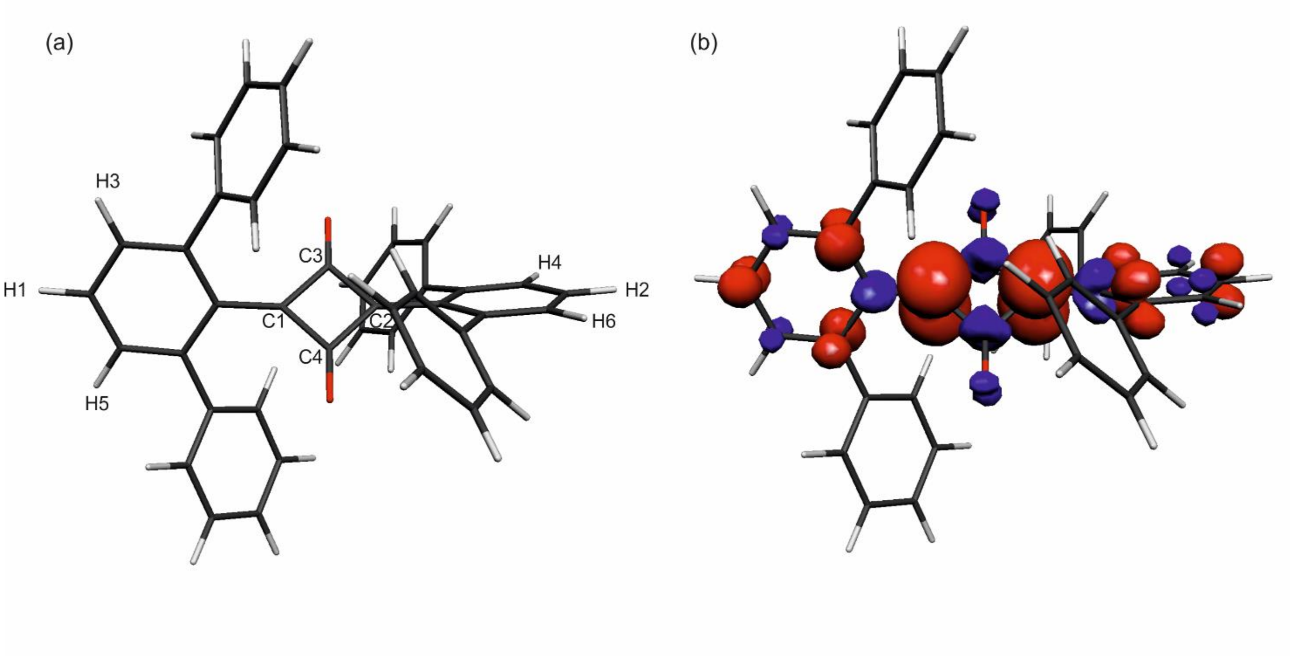

(a) Atom labeling for **2a•−**; (b) Spin density distribution of **2a•−** at the 0.003 eÅ^{-3} level.

Supplementary Figure 22: Optimised geometries for D^{Naph}, E^{Naph}, D^{Xyl}, and E^{Xyl}

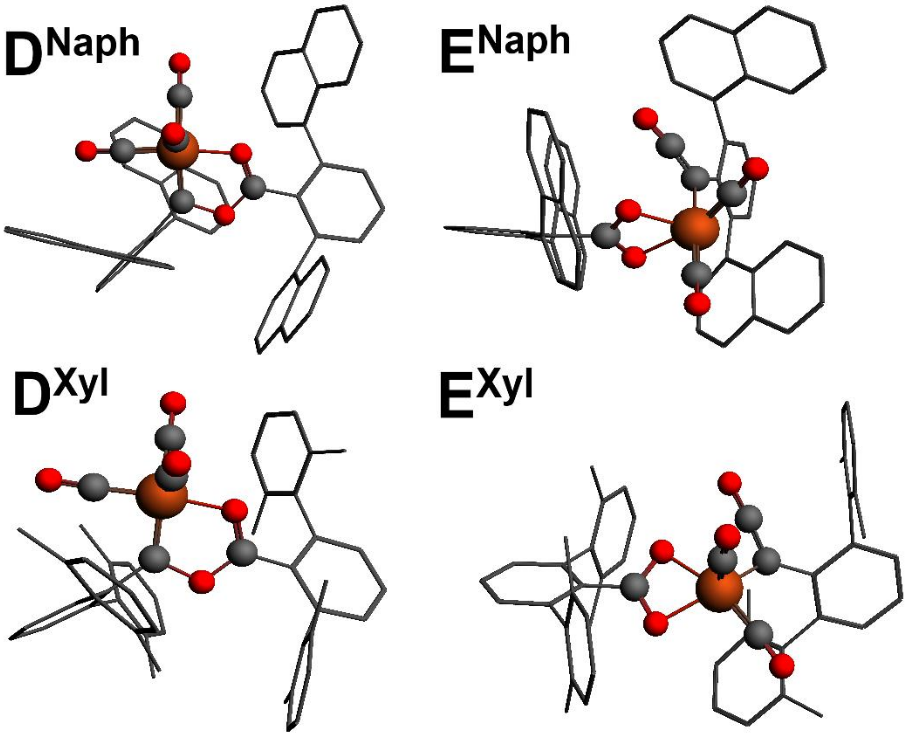

Optimised geometries for **D^{Naph}** (compound **4**), **E^{Naph}**, **D^{Xyl}**, and **E^{Xyl}**. Hydrogen atoms omitted and terphenyl framework represented as sticks for clarity.

Supplementary Tables

Supplementary Table 1: Bands (cm^{-1}) and isotopic shifts (cm^{-1}) observed in the reaction between 1^{Mes} and CO or ^{13}CO.

CO	^{13}CO	Isotopic Shift
2097	2038	–59
2050	2001	–49
2030	1982	–48
1978	1917	–61
1751	1705	–46

Supplementary Table 2: Parameters for the experimental and simulated EPR spectra for $2^{Mes•–}$, $2^{Mes•–}$-^{13}C and $2^{Xyl•–}$.

Compound	g_{iso}	$\lvert a_{iso}\rvert$ /10^{-4} cm^{-1}	Linewidth /G	Lineshape
$2^{Mes•–}$	2.0028	0.96 (2H)<br>0.86 (4H)<br>19.43 (2C)<br>11.12 (2C)	0.52	Gaussian
$2^{Mes•–}$-^{13}C	2.0028	0.96 (2H)<br>0.86 (4H)<br>19.43 (2C)<br>11.12 (2C)	3.60	Lorentzian
$2^{Xyl•–}$	2.0027	1.16 (2H)<br>0.96 (4H)[a]	0.23	Lorentzian

[a]Coupling to natural abundance ^{13}C was noted but not sufficiently resolved to be included in the simulation parameters.

Supplementary Table 3: Scaled calculated harmonic frequencies (cm^{-1}), from the DFT calculations. A scaling factor of 0.95 has been applied.

Neutral, ^{12}C	Neutral, ^{13}C	Reduced, ^{12}C	Reduced, ^{13}C
28.1580	28.1580	12.1790	12.1790
30.9035	30.8750	23.3320	23.3320
40.2990	40.2895	28.3575	28.3290
44.5550	44.5360	30.3145	30.3050
54.3210	54.1690	35.8150	35.7865
61.9495	61.9210	40.6410	40.4795
64.3340	64.2865	51.1955	51.1670
66.4430	66.3195	56.1450	56.0595
69.9960	69.9010	61.2370	61.1990
73.7200	73.6345	64.7425	64.6000
81.4720	81.4340	71.2215	71.0505
85.3385	84.9870	72.5705	72.2095
91.8175	90.9150	78.8595	78.3370
93.8695	93.5180	87.7325	87.3335
108.918	108.556	108.034	107.654
121.980	121.961	122.075	121.885
125.010	124.934	125.191	125.096
142.253	141.920	126.274	126.027
149.720	149.691	153.235	153.159
179.645	179.379	157.035	156.684
204.060	203.243	194.493	193.961
214.966	213.617	203.224	202.027
220.162	219.507	214.652	214.016
241.917	241.908	232.883	232.864
250.847	250.467	239.647	239.286
273.609	269.553	273.847	268.964
276.137	275.481	277.276	276.820
276.916	276.459	280.088	280.070
300.684	300.409	300.694	298.357
302.034	301.938	309.956	309.757
314.469	312.882	314.754	313.671
328.500	327.997	328.159	327.598
352.811	350.626	358.881	358.701
358.445	358.312	363.460	361.456
403.854	403.845	393.519	393.119
404.349	404.320	394.867	394.791
409.659	409.640	401.822	401.603
411.464	411.435	402.211	402.106
424.298	423.795	412.338	406.885
442.795	442.225	431.110	430.549
471.903	468.378	456.276	454.898
492.622	491.473	489.525	486.903
496.442	493.667	510.957	510.844
517.522	517.446	516.258	515.432
547.247	543.457	530.793	529.558
547.722	544.939	550.021	547.010
554.981	554.125	553.498	551.275
599.288	591.498	566.457	564.794
599.991	599.250	586.159	586.026
601.863	599.934	587.736	587.717
602.338	601.834	603.858	602.461
611.524	602.253	604.827	603.839
611.582	605.102	605.644	604.808
612.019	611.372	606.138	605.549
613.586	611.648	614.156	607.126
615.277	612.588	617.481	613.044
622.240	621.851	624.995	617.984
631.607	626.924	626.867	622.431
654.246	651.700	649.914	647.207

666.244	664.373	668.591	666.396
683.591	683.468	684.038	673.569
685.121	684.997	684.883	684.047
701.556	699.038	687.819	684.903
702.401	702.173	688.303	687.800
715.036	704.739	692.160	688.256
717.260	716.452	718.865	714.096
745.855	734.635	724.128	718.219
747.080	746.358	724.432	723.634
754.832	748.296	739.803	737.779
755.687	754.490	740.914	740.800
765.073	760.418	752.144	751.317
768.047	767.904	752.913	752.162
771.885	769.443	761.168	753.625
787.350	776.482	769.167	762.498
801.116	800.679	777.499	777.280
808.583	806.341	782.629	780.691
814.150	814.055	797.401	797.059
829.027	822.197	815.242	811.889
840.693	840.683	817.095	817.067
841.842	841.795	822.063	819.299
847.628	847.609	822.481	822.206
852.482	852.473	825.265	822.510
895.042	895.005	891.433	891.366
895.888	895.850	891.841	891.774
917.111	917.111	899.707	899.678
920.293	920.293	900.372	900.296
933.679	933.669	902.348	902.338
935.702	935.683	902.547	902.547
952.812	952.803	933.859	933.841
953.183	953.183	934.078	934.059
959.614	959.595	938.714	938.619
962.540	962.521	938.875	938.733
968.563	968.544	955.206	955.206
969.351	969.332	955.434	955.434
975.659	975.555	972.353	972.239
976.534	976.467	972.486	972.420
978.614	978.576	973.180	972.895
978.956	978.947	974.187	973.883
985.948	985.881	976.894	974.909
986.375	986.337	978.671	976.866
991.391	990.831	984.646	978.643
991.847	991.230	986.366	984.808
999.884	998.079	989.805	986.347
1001.64	999.884	990.043	988.969
1004.46	1001.65	1001.12	989.558
1021.05	1020.27	1006.86	994.916
1025.15	1024.75	1021.15	1006.38
1033.59	1026.06	1024.93	1011.87
1037.88	1033.97	1028.83	1026.27
1039.30	1034.48	1040.43	1028.08
1058.80	1040.32	1040.75	1040.11
1071.10	1046.10	1043.82	1041.77
1077.74	1075.50	1065.37	1064.97
1079.26	1076.63	1065.88	1065.61
1080.27	1078.44	1078.74	1075.79
1084.29	1079.22	1079.48	1078.60
1085.59	1083.54	1082.10	1079.96
1092.21	1083.58	1085.18	1083.34
1125.89	1118.10	1101.63	1099.66
1129.38	1123.44	1108.17	1103.88
1149.54	1149.52	1137.78	1137.78
1151.84	1151.83	1138.31	1138.30
1158.11	1158.11	1155.91	1155.84

1161.24	1161.23	1156.28	1156.19
1164.74	1164.71	1159.09	1158.48
1165.74	1165.70	1161.10	1159.91
1173.71	1172.95	1177.72	1177.72
1174.74	1174.00	1178.07	1178.06
1177.59	1177.57	1184.62	1184.62
1183.43	1183.42	1185.32	1185.32
1251.44	1251.34	1236.33	1236.12
1251.88	1251.86	1236.55	1236.52
1269.51	1266.28	1264.05	1252.87
1273.43	1272.68	1265.34	1264.62
1276.14	1273.08	1268.75	1265.10
1280.27	1279.99	1278.56	1278.19
1290.48	1289.53	1282.61	1280.80
1291.25	1290.52	1285.75	1285.55
1295.06	1291.62	1290.14	1288.32
1296.41	1296.35	1291.66	1291.63
1309.79	1297.55	1296.74	1293.19
1317.35	1316.65	1315.29	1315.28
1318.49	1318.44	1315.63	1315.60
1319.73	1318.82	1329.55	1329.48
1322.57	1322.49	1330.56	1330.50
1357.37	1337.66	1355.29	1339.35
1410.54	1407.88	1397.96	1396.09
1437.63	1428.50	1429.37	1422.99
1439.72	1438.08	1430.46	1429.79
1440.61	1440.45	1433.78	1430.41
1444.56	1444.30	1439.80	1439.56
1453.88	1448.64	1448.73	1444.23
1457.06	1456.86	1450.87	1450.77
1459.45	1458.61	1459.25	1452.33
1488.53	1488.45	1496.60	1496.59
1491.04	1490.95	1496.76	1496.72
1496.93	1496.89	1503.39	1503.37
1498.71	1498.67	1504.08	1504.05
1572.13	1571.99	1563.97	1563.84
1574.53	1574.30	1564.44	1563.98
1589.95	1589.91	1577.47	1577.44
1590.43	1590.4	1579.37	1579.13
1598.31	1598.3	1588.73	1588.72
1598.45	1598.43	1589.04	1588.99
1601.87	1601.85	1598.29	1598.29
1602.74	1602.72	1598.68	1598.61
1614.63	1614.63	1621.88	1621.45
1616.17	1616.17	1622.31	1622.30
1619.35	1619.34	1626.16	1623.26
1620.61	1620.61	1626.51	1626.17
1719.86	1675.18	1666.21	1626.55
1818.79	1768.50	1768.04	1719.00
3031.15	3031.15	3019.52	3019.52
3032.53	3032.53	3019.55	3019.55
3038.13	3038.13	3021.85	3021.85
3038.25	3038.25	3022.12	3022.12
3038.42	3038.42	3022.59	3022.59
3038.56	3038.55	3022.62	3022.62
3041.10	3041.10	3028.55	3028.55
3041.88	3041.88	3028.64	3028.64
3046.27	3046.27	3033.56	3033.56
3047.73	3047.73	3033.64	3033.64
3051.14	3051.14	3034.08	3034.08
3051.28	3051.28	3034.25	3034.25
3051.98	3051.98	3040.45	3040.45
3053.54	3053.54	3040.66	3040.66
3054.03	3054.03	3043.32	3043.32

3056.08	3056.08	3043.38	3043.38
3058.48	3058.48	3049.59	3049.59
3059.15	3059.15	3049.82	3049.82
3059.32	3059.32	3055.53	3055.53
3060.98	3060.98	3055.91	3055.91
3065.92	3065.92	3059.57	3059.57
3068.43	3068.43	3059.74	3059.74
3071.77	3071.77	3059.77	3059.77
3075.03	3075.03	3060.02	3060.02
3075.17	3075.17	3068.42	3068.42
3076.85	3076.85	3070.82	3070.82

Supplementary Table 4: Calculated isotropic hyperfine couplings from DFT calculations on 2a•−

Atom	a_{iso} /10^{-4} cm^{-1}
C1	15.4
C2	15.4
C3	-10.6
C4	-10.9
H1	-2.1
H2	-2.1
H3	1.6
H4	1.6
H5	1.4
H6	1.4

Supplementary Table 5: Geometry optimised coordinates of 2a.

C	-1.0153169181	-0.0542311819	-0.0259510911
C	-0.0001631205	-0.0002401721	-1.0904392837
C	0.0057472806	-0.0097021268	1.0354767154
C	1.0211740778	0.0479018830	-0.0307084772
C	-1.7156581731	2.7473237771	-0.9047190186
C	-2.5154232762	1.8382907367	-1.6053268485
C	-2.6670005411	1.9869977026	-2.9851898231
C	-2.0171607005	3.0167037091	-3.6564509675
C	-1.2140805967	3.9115427496	-2.9546381379
C	-1.0701463328	3.7792707834	-1.5773691622
C	-3.1969121022	0.7437407306	-0.8676466968
C	-2.4582309181	-0.1284072282	-0.0385898175
C	-3.1215567349	-1.0745362321	0.7729933276
C	-4.5122357384	-1.1335062771	0.7441685910
C	-5.2407189224	-0.2905543180	-0.0887382896
C	-4.5856261032	0.6379516858	-0.8908164328
O	-0.0043393494	0.0050268020	-2.3037782830
C	-2.3370495508	-2.0378091898	1.5866202002
C	-2.4874972877	-2.0881521652	2.9733872296
C	-1.7041651168	-2.9474461240	3.7344671002
C	-0.7730562068	-3.7773741074	3.1166939423
C	-0.6386544686	-3.7539431325	1.7321819164
C	-1.4175856399	-2.8912441736	0.9691070449
O	0.0090965095	-0.0170291009	2.2484737150
C	2.4634400721	0.1309569287	-0.0469897509
C	3.1247321965	1.0719089672	0.7721681045
C	4.5147421870	1.1418120107	0.7335428410
C	5.2439490536	0.3141240160	-0.1141902789
C	4.5908679310	-0.6119790220	-0.9208961362
C	3.2034049406	-0.7270950656	-0.8898458721
C	2.3385733238	2.0155789598	1.6070862326

C	2.5016715840	2.0479649944	2.9928362012
C	1.7209977041	2.8924689861	3.7731233305
C	0.7791575648	3.7249019434	3.1751994903
C	0.6304933043	3.7181459092	1.7920305185
C	1.4072921839	2.8706289174	1.0098363901
C	2.5189918357	-1.8176081032	-1.6306047200
C	2.6451875789	-1.9479981286	-3.0149067410
C	1.9797574846	-2.9666351641	-3.6877345939
C	1.1898156462	-3.8712481743	-2.9837044258
C	1.0746569031	-3.7595691487	-1.6020124064
C	1.7347359976	-2.7383031133	-0.9276355526
H	5.0260302809	1.8705550402	1.3554517291
H	-5.1583302427	1.3164466543	-1.5160733392
H	-6.3245039236	-0.3510613530	-0.1057370840
H	-5.0241205985	-1.8645912803	1.3626227030
H	5.1643098324	-1.2795950172	-1.5568832301
H	6.3270090466	0.3838250500	-0.1394374846
H	3.2379704511	-1.2277751214	-3.5707158680
H	2.0604492833	-3.0407831844	-4.7679026075
H	0.6636485716	-4.6601502024	-3.5127543098
H	0.4697740307	-4.4695231561	-1.0450802773
H	1.6534981990	-2.6613150929	0.1539184611
H	3.2121966942	1.3749480271	3.4639480816
H	1.8319709079	2.8873060126	4.8530433096
H	0.1591896597	4.3716299366	3.7880215935
H	-0.0930558059	4.3765088761	1.3187416409
H	1.2941009801	2.8679298908	-0.0715075886
H	-3.1884912174	-1.4161571772	3.4599773475
H	-1.8036999128	-2.9542941042	4.8155211190
H	-0.1488510732	-4.4340490748	3.7144748385
H	0.0751774604	-4.4117141201	1.2438107958
H	-1.3158948445	-2.8768351933	-0.1132799745
H	-3.2721896227	1.2755886715	-3.5388236939
H	-2.1206239064	3.1065866829	-4.7334899499
H	-0.7029777216	4.7116937547	-3.4817962512
H	-0.4537932506	4.4810218149	-1.0224012933
H	-1.6137529669	2.6560238033	0.1738999641

Supplementary Table 6: Geometry optimised coordinates of 2b

C	0.15716	-2.44140	0.46782
C	-0.45833	-3.37873	-0.42761
C	-0.28816	-4.74974	-0.20476
C	0.42440	-5.22044	0.90079
C	0.97192	-4.31182	1.81147
C	0.86109	-2.93133	1.61713
H	-1.80876	5.29314	0.22810
H	-0.76234	-5.45450	-0.89037
H	0.53788	-6.29371	1.06294
H	1.52079	-4.67371	2.68289
O	-0.45193	0.44353	2.14638
C	0.08083	-1.03266	0.19590
C	0.27490	-0.17543	-1.00973
C	0.03474	1.07025	-0.22880
C	-0.17268	0.21250	0.97217
C	0.02389	2.48347	-0.48838
C	1.06082	3.09569	-1.26681
C	1.02807	4.47792	-1.47771
C	0.01957	5.27307	-0.92465
C	-0.99751	4.68156	-0.17111
C	-1.03168	3.29922	0.03975
O	0.53082	-0.40714	-2.18849
H	1.82576	4.93659	-2.06515
H	0.02065	6.35180	-1.09114
H	-1.01999	-3.04558	-1.22672
H	1.28067	-2.27336	2.29244
H	1.82138	2.52385	-1.66627
H	-1.80721	2.87100	0.56910

Supplementary Table 7: Geometry optimised coordinates of 2a$^{\bullet-}$

C	-1.0181790496	0.1914900896	-0.0318358839
C	-0.0009458830	-0.0001703645	-1.0762566384
C	-0.0002272005	0.0011926349	1.0188663611
C	1.0170539670	-0.1899988224	-0.0322743936
C	-0.9942960438	3.1322706710	-0.9114992830
C	-1.9312198996	2.4046602758	-1.6549090779
C	-1.9721296977	2.5796226145	-3.0376711676
C	-1.1000676401	3.4608193506	-3.6700214558
C	-0.1712367843	4.1782557468	-2.9239276573
C	-0.1227769865	4.0123504084	-1.5414225708
C	-2.8920359660	1.5056575659	-0.9594707947
C	-2.4304990531	0.4839329997	-0.0920082374
C	-3.3785801385	-0.2319746749	0.6763749605
C	-4.7379281312	0.0599842034	0.5447846152
C	-5.1844310385	1.0301957597	-0.3447389152
C	-4.2583259563	1.7483694430	-1.0928871175
O	-0.0013756973	-0.0009599491	-2.3018996380
C	-2.9783502331	-1.3180312117	1.6111815392
C	-3.4715134310	-1.3189376098	2.9178384649
C	-3.1509145211	-2.3410341728	3.8047510010
C	-2.3256784137	-3.3825113330	3.3939496228
C	-1.8299842159	-3.3934889326	2.0918987031
C	-2.1533501258	-2.3733793696	1.2051851676
O	-0.0000023859	0.0010362187	2.2427463612
C	2.4293819889	-0.4820727902	-0.0932720403
C	3.3781288417	0.2345716167	0.6736177616
C	4.7374358745	-0.0568973878	0.5405341066
C	5.1833480513	-1.0274197928	-0.3489233629
C	4.2565851956	-1.7465761889	-1.0952851603
C	2.8903471646	-1.5044231838	-0.9602574828
C	2.9789116528	1.3202170458	1.6093311833
C	3.4742834550	1.3203796901	2.9151502583
C	3.1552962761	2.3419750999	3.8031817226
C	2.3294282929	3.3837418691	3.3943781006
C	1.8314954895	3.3954752268	2.0931860195
C	2.1532816683	2.3758258172	1.2053315547
C	1.9293773086	-2.4054625558	-1.6528491986
C	1.9691705258	-2.5833182135	-3.0353001077
C	1.0985116595	-3.4679605515	-3.6647858176
C	0.1720395772	-4.1858282346	-2.9161866156
C	0.1243283605	-4.0166325774	-1.5340287035
C	0.9943982272	-3.1330602406	-0.9069589932
H	5.4534507608	0.5119679235	1.1281589437
H	-4.5881478890	2.5387751089	-1.7633625444
H	-6.2481360323	1.2303386666	-0.4433181718
H	-5.4534701958	-0.5085035469	1.1333457780
H	4.5859573314	-2.5375144943	-1.7653517331
H	6.2470120751	-1.2271547942	-0.4486721065

H	2.6704955898	-2.0002514643	-3.6247252796
H	1.1281488279	-3.5743540660	-4.7459417624
H	-0.5173693186	-4.8661784997	-3.4092863955
H	-0.5960197056	-4.5720803247	-0.9387125477
H	0.9508960581	-2.9975897286	0.1702529356
H	4.1013074419	0.4933828713	3.2379927478
H	3.5413511235	2.3140076010	4.8186747945
H	2.0708581532	4.1787121892	4.0891126822
H	1.1811355034	4.2049440445	1.7702005349
H	1.7572938209	2.3890493178	0.1943254884
H	-4.0979355160	-0.4921024822	3.2422679756
H	-3.5351256762	-2.3136867047	4.8209579297
H	-2.0658604843	-4.1778319908	4.0878120415
H	-1.1800561316	-4.2026980592	1.7674021876
H	-1.7589959720	-2.3859898350	0.1935262333
H	-2.6751015834	1.9966123037	-3.6252069960
H	-1.1303934808	3.5647308335	-4.7513875099
H	0.5196222615	4.8553335404	-3.4194678756
H	0.5993618995	4.5674657228	-0.9479537262
H	-0.9500532013	2.9991461869	0.1659597872

Supplementary Table 8: Geometry optimised coordinates of D^{Naph} (compound 4)

Fe	-0.0628036348	-1.0088489487	-1.4333351751
O	0.0909772069	0.2157463910	0.9977104587
O	1.0565230926	0.5780843350	-0.9619003617
O	-1.2741160970	0.4822779886	-3.6858085646
O	-1.7351942544	-3.3986845195	-1.6272486536
O	2.2788959791	-2.3507647903	-2.6747256603
C	-0.5871048841	-0.8511565836	0.2747726424
C	0.9415919770	0.9083786775	0.2485821628
C	-0.7779937226	-0.1202299594	-2.8233349983
C	-1.0805911077	-2.4344120656	-1.5721783076
C	1.3620318608	-1.8060923020	-2.2102201684
C	-1.5630971284	-1.4271823436	1.2347499580
C	-1.1137584576	-2.1695082269	2.3474574832
C	-2.0465847651	-2.7072697619	3.2384021685
H	-1.6917748752	-3.2836891576	4.0860772640
C	-3.4128821551	-2.5118080836	3.0395446234
H	-4.1285572850	-2.9307708748	3.7382688111
C	-3.8555687312	-1.7705530605	1.9487658233
H	-4.9156856237	-1.5958360814	1.7984750336
C	-2.9440934933	-1.2157643632	1.0399130426
C	0.3445776792	-2.3905933307	2.5942975408
C	0.9917289463	-1.6597537311	3.5687744800
H	0.4311446457	-0.9249013262	4.1366531549
C	2.3710554727	-1.8418270095	3.8285616465
H	2.8515791617	-1.2496955717	4.5995589535
C	3.0955367211	-2.7582316576	3.1043667446
H	4.1557864216	-2.8990599121	3.2920892550
C	2.4678224914	-3.5392131921	2.0956422455
C	3.1960551034	-4.4939307946	1.3303187628
H	4.2550093843	-4.6258390321	1.5340800988
C	2.5774450794	-5.2344170995	0.3524561739
H	3.1451962075	-5.9482364856	-0.2337927676
C	1.1893923380	-5.0759961326	0.1135221609
H	0.7024713852	-5.6731146611	-0.6493214524
C	0.4572416731	-4.1744837998	0.8474307640
H	-0.6074381775	-4.0721227195	0.6768027279
C	1.0749475344	-3.3651294391	1.8409452261
C	-3.4313016041	-0.3457244928	-0.0698570624
C	-3.0277658237	0.9735693041	-0.1037592556
H	-2.3725359991	1.3404650527	0.6760583482
C	-3.4380739589	1.8470913485	-1.1343849334
H	-3.0931831312	2.8749800516	-1.1270394628
C	-4.2598344867	1.3880742951	-2.1342882172
H	-4.5742376973	2.0468912482	-2.9375464652
C	-4.6987425140	0.0361967178	-2.1462940428
C	-5.5339736102	-0.4584020232	-3.1868911676
H	-5.8417309336	0.2260341504	-3.9715336094
C	-5.9340221552	-1.7721193845	-3.2085964477
H	-6.5671369711	-2.1380402422	-4.0094307401
C	-5.5084509498	-2.6579654236	-2.1880032916
H	-5.8108624464	-3.6988847079	-2.2212559956
C	-4.7055032693	-2.2111186865	-1.1659618942
H	-4.3679144254	-2.9037520116	-0.4044067507
C	-4.2830035813	-0.8513758513	-1.1051060791
C	1.7076887400	1.9907244212	0.8883584442
C	3.0950880011	2.0450560747	0.6395415925
C	3.8369506089	3.1074837975	1.1634803897
H	4.9046144639	3.1418571115	0.9782390108

C	3.2176449649	4.0969361179	1.9225099039
H	3.8005885621	4.9187606388	2.3226258286
C	1.8519308556	4.0228358429	2.1837406201
H	1.3652182598	4.7867772869	2.7794820641
C	1.0777669941	2.9684228306	1.6849870605
C	3.7868636101	0.9455854698	-0.0937021825
C	3.8328341518	-0.3107364612	0.4757440858
H	3.3605674937	-0.4848833684	1.4381824046
C	4.4868906144	-1.3818008931	-0.1751121807
H	4.4884022307	-2.3601223413	0.2890555932
C	5.0903332407	-1.1841973121	-1.3916591240
H	5.5824263349	-2.0051447489	-1.9027977632
C	5.0543623996	0.0903305031	-2.0194621621
C	5.6464813357	0.3043741037	-3.2951300402
H	6.1453970163	-0.5285601809	-3.7807139229
C	5.5790633251	1.5318744624	-3.9078048102
H	6.0295014596	1.6813203059	-4.8828750659
C	4.9078744984	2.6054721687	-3.2717646940
H	4.8463142733	3.5672644726	-3.7690108517
C	4.3281436978	2.4317676535	-2.0374885638
H	3.8021417460	3.2542213730	-1.5665368392
C	4.3916163923	1.1756486873	-1.3702832022
C	-0.3819709984	2.9576004957	2.0140129772
C	-0.8542397255	2.1432048323	3.0216036245
H	-0.1715961821	1.4596788318	3.5120439548
C	-2.2139222049	2.1712363446	3.4095966571
H	-2.5566989652	1.5028905812	4.1907755156
C	-3.0888380565	3.0338909840	2.7957245571
H	-4.1337624945	3.0625568517	3.0873213638
C	-2.6479301993	3.8868518717	1.7476398121
C	-3.5453623560	4.7635452184	1.0770721181
H	-4.5828309722	4.7865434769	1.3953713461
C	-3.1166583672	5.5535250450	0.0378201312
H	-3.8106070581	6.2130455905	-0.4709191251
C	-1.7644323998	5.4985396017	-0.3840694997
H	-1.4375925979	6.1147233580	-1.2142316581
C	-0.8696467265	4.6670164889	0.2476150166
H	0.1616140317	4.6255664639	-0.0846158474
C	-1.2803652677	3.8435150154	1.3350200186

Supplementary Table 9: Geometry optimised coordinates of E^{Naph}

Fe	0.2605999428	-0.4507355530	1.3991995727
O	1.7068225573	-0.3448204275	0.0626262361
O	0.9434762252	1.4862154921	0.9804127438
C	-0.6615702008	-0.7073122440	-1.2058496986
O	-0.1812434486	-3.3773415249	1.1913461271
O	2.5400957303	-0.5390013650	3.3806153053
C	-1.1635733937	-0.3653202650	-0.0444567106
C	1.7488133409	0.9560161154	0.1302508448
O	-0.1211265921	-1.0024836319	-2.2293686606
C	-0.0309683635	-2.2374154377	1.3093145708
C	1.6236949503	-0.5149947835	2.6808359001
C	-2.5342682838	0.1476741908	0.1140975904
C	-2.8359094551	1.0406020880	1.1690697327
C	-4.0890874136	1.6573798104	1.2456599040
H	-4.2799380755	2.3510691850	2.0581581476
C	-5.0801057080	1.3849455743	0.3085990802
H	-6.0470003189	1.8709193685	0.3724596625
C	-4.8252574222	0.4466295875	-0.6876401710
H	-5.6009313855	0.1797619368	-1.3981808571
C	-3.5827893137	-0.1861216571	-0.7886479396
C	-1.8454376814	1.3587815854	2.2319247783
C	-1.3815910111	2.6455651980	2.3857420879
H	-1.6817693068	3.3992588334	1.6705661177
C	-0.4771853102	2.9886771613	3.4152017551
H	-0.1019711734	4.0044554667	3.4647043963
C	-0.0683981981	2.0514034424	4.3306577256
H	0.6180315601	2.3129554916	5.1293247570
C	-0.5494620041	0.7169981062	4.2504132586
C	-0.2080279255	-0.2597620114	5.2241696915
H	0.4612122413	0.0252159530	6.0296604274
C	-0.7140801976	-1.5388341675	5.1599267674
H	-0.4487294674	-2.2679402866	5.9169821347
C	-1.5962976685	-1.9028168807	4.1176777121
H	-2.0131066267	-2.9031093894	4.0873197531
C	-1.9380641689	-0.9881756086	3.1440484850
H	-2.6451347626	-1.2528280789	2.3674136536
C	-1.4159173063	0.3453132837	3.1635657563
C	-3.4026069461	-1.2078357003	-1.8616062187
C	-3.4625897012	-0.8398970259	-3.1899898024
H	-3.6499181274	0.1981155642	-3.4413294967
C	-3.2589141100	-1.7796223067	-4.2283902513
H	-3.2922343888	-1.4481491767	-5.2603434223
C	-3.0010400185	-3.0931503666	-3.9282695715
H	-2.8202359135	-3.8175951021	-4.7161898155
C	-2.9811192657	-3.5329822680	-2.5759927793
C	-2.7637663802	-4.8990037468	-2.2431375121
H	-2.5782314950	-5.6063246103	-3.0456590309
C	-2.7926107738	-5.3210920852	-0.9361051146
H	-2.6232534689	-6.3644193440	-0.6941533046
C	-3.0526436649	-4.3925390121	0.1011624594
H	-3.0894678901	-4.7355317420	1.1293582324
C	-3.2508297603	-3.0644192999	-0.1891096179
H	-3.4633219216	-2.3559294611	0.6026533965
C	-3.2058348425	-2.5908216913	-1.5291531373
C	2.6748411516	1.7292455247	-0.7170630499
C	3.8898474273	1.1428751654	-1.1326768894
C	4.7600930504	1.8790877364	-1.9432467278
H	5.6909592019	1.4217421587	-2.2585564821

C	4.4443166858	3.1775162545	-2.3315449818
H	5.1250120407	3.7384692661	-2.9621175672
C	3.2542978276	3.7587658580	-1.9032490398
H	2.9990508237	4.7696393761	-2.2005792654
C	2.3583734414	3.0524188670	-1.0936347138
C	4.3472786380	-0.2050812745	-0.6662394346
C	5.1337752900	-0.2799838031	0.4631170385
H	5.3777732211	0.6281249150	1.0053325423
C	5.6229367570	-1.5244269725	0.9308772792
H	6.2327985997	-1.5533226127	1.8271017407
C	5.3254773270	-2.6796803941	0.2523355754
H	5.6975217629	-3.6378007984	0.6027495147
C	4.5185473018	-2.6448887881	-0.9195162146
C	4.1760275779	-3.8328435189	-1.6230685608
H	4.5614283768	-4.7811838812	-1.2604285216
C	3.3623221835	-3.7852917554	-2.7295587884
H	3.0954945322	-4.6979370587	-3.2512534761
C	2.8527344330	-2.5442465289	-3.1849421925
H	2.1827123676	-2.5173019206	-4.0360299858
C	3.1786854045	-1.3787770464	-2.5349937023
H	2.7584329302	-0.4375757862	-2.8685103435
C	4.0196615088	-1.3944298438	-1.3899259357
C	1.1042181495	3.7585684927	-0.6874801445
C	1.1840982213	4.8075806704	0.2029126775
H	2.1435495222	5.0591871297	0.6423240600
C	0.0356714840	5.5545296372	0.5588486532
H	0.1323839916	6.3785594631	1.2576376478
C	-1.1869872372	5.2402458175	0.0179675636
H	-2.0734274806	5.8088424484	0.2837280362
C	-1.3145535550	4.1643587308	-0.9046394879
C	-2.5728346636	3.8133883642	-1.4661831072
H	-3.4553140070	4.3619616046	-1.1519904227
C	-2.6736586024	2.7989729329	-2.3865837575
H	-3.6412417951	2.5359797938	-2.7991260366
C	-1.5178923497	2.0854410696	-2.7851240317
H	-1.5970249063	1.2813263893	-3.5070115569
C	-0.2930553500	2.3818674920	-2.2384204953
H	0.5762163229	1.8072965805	-2.5376810470
C	-0.1553462949	3.4197526058	-1.2775514524

Supplementary Table 10: Geometry optimised coordinates of D^{Xyl}

Fe	-0.3276211090	-1.5781874054	-1.5042997206
O	0.0248626189	0.3518198870	0.4212397352
O	0.9570380613	-0.0878069127	-1.5425095445
O	-1.7339206082	-0.6980299767	-3.9437762505
O	-1.9177492800	-3.9197874306	-0.7606099228
O	1.7007827927	-3.4021476426	-2.6938674249
C	-0.7810418135	-0.8176283213	0.0647198205
C	0.9505814885	0.6292115138	-0.5054714255
C	-1.1658510043	-1.0625778329	-2.9953477585
C	-1.3181871718	-2.9710296541	-1.0787617653
C	0.9244851046	-2.6678375974	-2.2350506481
C	-1.8196735278	-1.1040048749	1.0892937001
C	-1.4743063165	-1.6498678769	2.3411530368
C	-2.4828313000	-2.1447571601	3.1802306271
H	-2.1954624213	-2.5716529433	4.1355280294
C	-3.8209399768	-2.0870086908	2.8078658852
H	-4.5885775582	-2.4829763710	3.4632815854
C	-4.1681979708	-1.4813665740	1.6036219858
H	-5.2109439835	-1.3673819015	1.3267826681
C	-3.1883102000	-0.9789328810	0.7421234074
C	-0.0798074054	-1.6888047071	2.8890839590
C	0.3510152378	-0.5923161591	3.6660172117
C	-1.9421849607	5.1385485080	0.6856525019
C	1.5670992767	-0.6804234928	4.3512671632
H	1.8972183330	0.1587813391	4.9557951467
C	2.3364338968	-1.8405085247	4.2884514838
H	-3.3382606023	1.3299355668	1.8284225855
C	1.9134703599	-2.9110395530	3.5056976575
C	-0.4964427113	3.8112172663	-0.7493451298
C	3.6655376142	-0.8780679945	1.3629003637
C	-1.1644102558	4.8036286669	1.7905156109
H	-1.4355273051	5.1674184189	2.7765827949
C	0.3297903001	3.5397123603	0.3624800171
C	3.9510289062	-0.2985200666	-2.4125447826
C	0.8436410960	3.7264605053	2.8540097703
C	-0.2112235807	3.2473917154	-2.1222319853
C	0.7122210517	-2.8483770422	2.7888669211
C	-3.6303595271	-0.1721107184	-0.4387510288
C	-3.5125769331	1.2322810350	-0.3278615185
C	4.6600871265	-2.5625558550	-1.9039472505
C	-3.9285151268	2.0362370507	-1.3916480016
H	-3.8422925690	3.1135586718	-1.3007688043
C	-4.4717619493	1.4712554853	-2.5440268795
H	-3.3410331325	2.9025496674	1.0145085824
C	-4.6372426840	0.0938914242	-2.6219165436
H	-1.9027925457	1.8880630181	0.9685352927
C	4.4279641170	-1.5444444221	-2.8272421298
C	-1.6139812364	4.6341841075	-0.5691690552
C	3.6481414362	0.7845791345	-3.4189310483
C	-0.5020772218	0.6479788668	3.7800882081
C	0.2942784323	-4.0046328373	1.9145087821
C	-4.4698070435	-2.2296803311	-1.7167249206
H	-3.8697277099	-2.6441769462	-2.5357712547
C	-4.2337443386	-0.7438171355	-1.5731710988
C	1.9855694105	1.6526313153	-0.2749871260
C	3.3204094622	1.2759863495	-0.5612307828
C	4.3473836556	2.2019918454	-0.3588598401
H	5.3669410588	1.8958575643	-0.5646724606

C	4.0704372083	3.4896049508	0.0875670723
H	4.8727902982	4.2045839856	0.2302342911
C	2.7545402668	3.8610829590	0.3301435482
H	2.5223801950	4.8750632307	0.6356185011
C	1.6943524883	2.9587800197	0.1607229790
C	3.7215765923	-0.0844844861	-1.0403260329
C	3.9401371694	-1.1069610065	-0.1017671572
C	-0.0171735683	4.0172414045	1.6444878822
C	4.4089876993	-2.3466773551	-0.5524605137
H	4.5793374302	-3.1409289742	0.1673664090
C	-2.9975472921	1.8700835250	0.9404827279
H	-5.5202532216	-2.4264597257	-1.9581761863
H	2.5167621003	-3.8118109351	3.4462223704
H	4.0310284454	-1.7133977831	1.9625275421
H	2.5912680727	-0.7873365108	1.5645630619
H	4.1397077570	0.0393390605	1.7282553572
H	1.6382667153	4.4728678890	2.9714693757
H	1.3234821022	2.7466279035	2.7855864816
H	0.2381883341	3.7455172279	3.7647046239
H	0.8596386192	3.1962143488	-2.3391512561
H	-0.6875019970	3.8557800075	-2.8957262558
H	-0.6204902141	2.2334605571	-2.2165058252
H	-5.0808130230	-0.3500597894	-3.5078063520
H	4.6027715726	-1.7218163629	-3.8835228594
H	-2.2268856977	4.8820431426	-1.4298673399
H	4.2183485452	1.6989752811	-3.2212126399
H	2.5839980825	1.0453377401	-3.3805106266
H	3.8813618749	0.4526095559	-4.4341507636
H	-0.0679269308	1.3550028990	4.4920114478
H	-1.5169477414	0.4067610825	4.1137443941
H	-0.5931239722	1.1476225214	2.8094937177
H	0.8349453376	-4.9167076131	2.1823821192
H	0.5148496480	-3.7778931398	0.8647101494
H	-0.7785322423	-4.2052003868	1.9845641715
H	-4.2238870569	-2.7822122880	-0.8099128569
H	3.2679437040	-1.9045157098	4.8409102878
H	-2.8109101793	5.7770748595	0.8045673964
H	5.0201382639	-3.5273830014	-2.2430268171
H	-4.7849921081	2.1052714746	-3.3665761034

Supplementary Table 11: Geometry optimised coordinates of E^{Xyl}

Fe	0.2935711310	-0.8184338271	-0.2825530615
O	-1.6848008499	-0.9369828237	-0.7121086840
O	-0.8898699254	0.5488822842	0.6844646128
H	3.7679814488	-2.5920044021	0.2535816518
O	1.4803653112	-3.1697997406	-1.6494391879
O	0.1733340014	-2.4569937740	2.1744052455
C	2.1174904863	-0.1632615831	0.2172009558
C	-1.9337829120	-0.0121673202	0.1592875060
H	5.1974511916	-3.4683792752	0.8289296226
C	1.0157767550	-2.2249188721	-1.1717632326
C	0.1860733478	-1.8346896127	1.2033932964
C	3.0959223459	0.1505827211	-0.8285513508
C	2.6370698840	0.4232897880	-2.1371188256
C	3.5524124121	0.7379767257	-3.1478727555
H	3.1814399393	0.9672507894	-4.1417489603
C	4.9190265058	0.7795882049	-2.8881593665
H	5.6198755190	1.0225301160	-3.6786536144
C	5.3758890552	0.5173423996	-1.5978716782
H	6.4375692724	0.5525885892	-1.3747832782
C	4.4854962580	0.2087420745	-0.5667775317
C	1.1718462789	0.4658724938	-2.4553127154
C	0.3732721296	1.5248467495	-1.9381360448
C	-1.4814544912	5.2112025593	0.2707475640
C	-0.9443828907	1.6656057499	-2.3963475635
H	-1.5305042996	2.4889986434	-2.0112474682
C	-1.4837485068	0.7901043165	-3.3271138262
C	2.2195049241	0.0772487812	1.4979606125
C	-0.6986426998	-0.2332862696	-3.8547732459
C	-1.9999591937	3.2780602114	1.6508303233
C	-4.5266375313	-2.1796196297	-1.7522127923
C	-2.3806804343	4.7710894938	-0.6978626073
H	-2.5301092688	5.3494028719	-1.6044130547
C	-2.8933981080	2.8312382003	0.6582002789
C	-3.1541924702	-2.8544658453	1.7572111055
C	-4.0812836043	3.1228552109	-1.5696387230
C	-1.7891534617	2.4986899825	2.9248982191
C	0.6318301299	-0.3881966716	-3.4652771120
C	5.0138546072	-0.0354055786	0.8084376264
C	5.3033690748	1.0620483225	1.6432208272
C	-2.9271909541	-4.8683185979	0.4224231333
C	5.8029396541	0.8237680677	2.9274416700
H	6.0193291995	1.6646917727	3.5785962917
C	6.0069456321	-0.4767972470	3.3811145396
H	5.2528068240	3.1946311289	1.9676256253
C	5.7102018905	-1.5576363230	2.5538514841
H	3.9967617740	2.6009016894	0.8690877741
C	-2.7390985105	-4.1848495279	1.6212624386
C	-1.2980610299	4.4697286173	1.4351860931
C	-2.9523204105	-2.1203482047	3.0644584503
C	0.9127273373	2.5580555073	-0.9797955011
C	1.4773891590	-1.4134044926	-4.1896982596
C	4.8550075697	-2.5290247783	0.3861825805
O	2.1488861569	0.2537397707	2.6795723604
C	5.2104168491	-1.3537906003	1.2642871651
C	-3.3112632919	0.2935621219	0.5998827716
C	-4.1927636960	-0.7919262623	0.8078725924
C	-5.4904718484	-0.5441117873	1.2631238112
H	-6.1538427393	-1.3856020541	1.4303843183

C	-5.9193688774	0.7557667675	1.5161333182
H	-6.9280514739	0.9381266488	1.8699228511
C	-5.0437261205	1.8192906146	1.3249990073
H	-5.3632422101	2.8361908442	1.5259264841
C	-3.7342207298	1.6102531646	0.8737648206
C	-3.7560504887	-2.2158907626	0.6570654093
C	-3.9483327339	-2.8964819242	-0.5579368887
C	-3.0997281946	3.5847640694	-0.5147304759
C	-3.5320175348	-4.2266516212	-0.6576016571
H	-3.6698910217	-4.7567191725	-1.5948733985
C	5.0428899216	2.4744107796	1.1724345552
H	5.6600611909	2.7328576537	0.3050873123
H	-1.1118833843	-0.9058983485	-4.5995586415
H	-4.7180765523	-2.8755851000	-2.5737093348
H	-3.8139259280	-1.4215730345	-2.0981051791
H	-5.4646126548	-1.6695116967	-1.5089005581
H	-5.1176728338	3.2545225081	-1.2378641487
H	-3.9548074493	2.0583554886	-1.7963074576
H	-3.9515308399	3.6896846322	-2.4961075483
H	-2.7352319720	2.1275478227	3.3326130739
H	-1.3071426104	3.1177165798	3.6863276212
H	-1.1481687815	1.6341609256	2.7265373852
H	5.8610068126	-2.5717916555	2.9106820456
H	-2.2666432436	-4.6808836008	2.4631489969
H	-0.6023536076	4.8142899553	2.1935591481
H	-3.9114768317	-1.8338555510	3.5107050534
H	-2.3795030257	-1.1952289394	2.9308646489
H	-2.4131773140	-2.7445289155	3.7811104031
H	0.4047237771	3.5131807010	-1.1374495897
H	1.9887062186	2.7030576439	-1.1043002962
H	0.7220887367	2.2584837668	0.0552220830
H	1.8374529914	-1.0009394120	-5.1397575654
H	0.8888367908	-2.3063485205	-4.4212595376
H	2.3582842550	-1.7162811042	-3.6224091039
H	5.2968848784	-2.4368243074	-0.6112981924
H	-0.9297907057	6.1333352358	0.1223500383
H	-2.5104843279	0.9116092535	-3.6555189103
H	-2.6040446525	-5.8997499507	0.3291781870
H	6.3885797382	-0.6484072238	4.3818572634

Supplementary Table 12: Geometry optimised coordinates for the transition state calculation for D^{Xyl} → E^{Xyl}

C	3.4947949729	3.4515119890	-1.6709655609
C	3.2612487042	4.4086603008	-0.6872006149
C	3.2086625659	4.0302502027	0.6542315840
C	3.4049391515	2.6971737111	1.0270652061
C	3.6398376184	1.7365818383	0.0240825106
C	3.6742615246	2.1041522551	-1.3329300312
C	3.3498936563	2.2849123287	2.4780464019
C	3.9036305675	0.3193185441	0.4209712150
C	2.8884911427	-0.6525497532	0.4569955102
C	3.1857611605	-1.9651311490	0.8591673713
C	4.5005704467	-2.2964399053	1.2067787656
C	5.5150747874	-1.3448674668	1.1477062412
C	5.2128795670	-0.0425659700	0.7601349073
C	1.5043358932	-0.2877739733	-0.0146156207
O	0.8915993551	0.6196892959	0.7130158472
Fe	-0.6343708579	1.7134836550	0.0415103779
C	0.2697016254	2.2331399105	-1.4559496240
O	0.6753532222	2.4515225754	-2.5173859346
C	2.1263673647	-3.0100341132	0.9966975006
C	1.4401000588	-3.1234141362	2.2200996150
C	0.4742579447	-4.1260781164	2.3621465742
C	0.1984934784	-5.0004698789	1.3130040296
C	0.8934297452	-4.8875062861	0.1102443341
C	1.8648199931	-3.8958102944	-0.0642047029
C	1.7523108924	-2.1818925351	3.3606060022
C	3.8634705285	1.0608393023	-2.4099429262
C	2.6054077374	-3.7501687038	-1.3702781373
C	-1.6749618444	0.4302049334	-0.1923429623
C	-2.6629353616	-0.5593470302	-0.4170993327
C	-2.7941361984	-1.2217099239	-1.6697820217
C	-3.7785517290	-2.2020371987	-1.7984412555
C	-4.6211020954	-2.5123565202	-0.7304910651
C	-4.5252076340	-1.8283513518	0.4834464362
C	-3.5595781107	-0.8411461296	0.6559697748
C	-1.9363774286	-0.8603515520	-2.8350162130
C	-0.8800440730	-1.7098248098	-3.2078784365
C	-0.0685084652	-1.3346099004	-4.2829250712
C	-0.3080209751	-0.1554184510	-4.9813972423
C	-1.3840059488	0.6571548918	-4.6296708738
C	-2.2148433561	0.3158483626	-3.5593159512
C	-3.5048401068	-0.0200604300	1.9028339221
C	-2.5226796748	-0.2740534451	2.8754496819
C	-2.4654026743	0.5519978725	4.0028630069
C	-3.3550901315	1.6124166401	4.1558220132
C	-4.3224259416	1.8573101290	3.1828269684
C	-4.4132735683	1.0475883685	2.0461890588
C	-0.6016879494	-2.9779271433	-2.4441789174
C	-3.4088348413	1.1781892396	-3.2199155436
C	-1.5064536692	-1.3681333352	2.6791908661
C	-5.4503380708	1.3383905676	0.9846534116
C	-0.0594303321	2.7290616640	1.4741999497
O	0.2674868686	3.2743307976	2.4415195950
C	-2.0247310965	2.7882900626	-0.2047007424
O	-2.9459081958	3.4897993796	-0.3239347202
O	1.0292263840	-0.7982702292	-1.0520085336
H	-3.8852678939	-2.7146045531	-2.7476605555
H	-5.3771164120	-3.2804483246	-0.8543367012

H	-5.2080050117	-2.0506577207	1.2954018325
H	0.7658663481	-1.9711175875	-4.5581766810
H	-1.9385710525	-2.2674489816	2.2308538538
H	0.6805180966	-5.5708068461	-0.7061779396
H	-1.7069841496	0.3649026154	4.7563243269
H	-1.0497271603	-1.6500112009	3.6308562317
H	-0.7032696231	-1.0266237701	2.0139361139
H	-6.2051041510	0.5458802269	0.9279286545
H	5.9902508021	0.7130670168	0.7133605629
H	6.5327636054	-1.6142970764	1.4083384036
H	4.7157086330	-3.3116819411	1.5242402129
H	3.5179848295	3.7414083218	-2.7165092530
H	-4.9967467084	1.4195989516	-0.0096248407
H	-1.5832930863	1.5658759356	-5.1884528239
H	3.9377686853	1.5294894152	-3.3949191331
H	3.0156431422	0.3654912620	-2.4302091533
H	4.7676260137	0.4663864156	-2.2399736580
H	3.6895404945	-3.8101304092	-1.2251456665
H	2.3827297167	-2.7710550881	-1.8100622702
H	2.3127963325	-4.5306595002	-2.0785591575
H	2.8128874079	-2.2250563814	3.6311220049
H	1.1640935491	-2.4340160907	4.2475591206
H	1.5334955735	-1.1431520711	3.0868307137
H	-5.0117102350	2.6873371324	3.3000388135
H	3.0179743854	4.7734989238	1.4222580550
H	-0.0600742301	-4.2185089538	3.3028255555
H	4.3083219160	1.8749978102	2.8156945944
H	2.5932629399	1.5061935589	2.6217753993
H	3.0952196365	3.1338753276	3.1176716456
H	0.2143152409	-3.5348685334	-2.9099794825
H	-1.4773697971	-3.6341683474	-2.3971303658
H	-0.2959918757	-2.7397872509	-1.4214542122
H	-4.3368370819	0.5945465877	-3.2270004071
H	-3.5182729334	1.9921512793	-3.9412622909
H	-3.3243743313	1.6276019853	-2.2261932090
H	-5.9641724722	2.2799835149	1.1932800962
H	0.3390369521	0.1287927819	-5.8040060605
H	-0.5522968881	-5.7740788280	1.4364436922
H	3.1173817767	5.4480088395	-0.9635966449
H	-3.2940988552	2.2491603024	5.0316906943

Supplementary Table 13. Geometry optimised coordinates for the transition state calculation for D^{Naph} → E^{Naph}

Fe	-0.1668088297	1.8789943680	-0.4390044908
O	0.0014868676	-0.7516963562	0.6252902796
O	-1.6390231829	0.6110210514	-0.0288735643
C	0.9267473586	0.3563112183	0.2469227400
C	-1.2848883326	-0.5240540446	0.3989109321
O	1.0722454974	-0.0407568436	-2.2621136120
O	1.9787596972	3.8402844003	-0.8341636744
O	-1.7642965340	3.1044873765	-2.6094655262
C	0.8363483476	0.5218192079	-1.2115321368
C	1.1500710192	3.0320206145	-0.6922889968
C	-1.1558827790	2.6414616640	-1.7314610930
C	2.0259696505	0.4732528851	1.2351913994
C	1.6984005935	0.6419709392	2.6108695789
C	2.7118995519	0.7989827742	3.5572178956
H	2.4380362830	0.9253236031	4.5995819248
C	4.0522327835	0.8205351307	3.1742248545
H	4.8309837713	0.9580518557	3.9158582717
C	4.3782376714	0.6556471059	1.8353660766
H	5.4167661266	0.6481090870	1.5216639991
C	3.3852771658	0.4627512469	0.8613197556
C	0.2826795800	0.6218603364	3.0893442502
C	-0.1500630132	-0.3870760174	3.9294167419
H	0.5641080171	-1.1302255947	4.2651224908
C	-1.5101700327	-0.5026821648	4.2914016581
H	-1.8224698369	-1.3150604545	4.9388087665
C	-2.4395974320	0.3837140776	3.7948862729
H	-3.4934151117	0.2703151971	4.0293437419
C	-2.0303420834	1.4535299752	2.9593746811
C	-2.9686246602	2.3746512534	2.3964586749
H	-4.0192140779	2.2500879504	2.6340023974
C	-2.5670953335	3.3781584657	1.5605599743
H	-3.2942631141	4.0465328308	1.1150956432
C	-1.1793969918	3.5712520078	1.2930350029
H	-0.8596612200	4.4584995851	0.7582993686
C	-0.2419018100	2.7093987474	1.8376533888
H	0.8166740981	2.9384857199	1.8038102083
C	-0.6535440832	1.5982006293	2.6447091102
C	3.8263684402	0.1790026478	-0.5354772779
C	3.5877724947	-1.0674185682	-1.0742106135
H	3.0305204313	-1.7979215902	-0.4980801425
C	4.0327723658	-1.4011248480	-2.3721306061
H	3.8120249773	-2.3855317454	-2.7658385085
C	4.7193791609	-0.4836216949	-3.1259022527
H	5.0587154246	-0.7311004679	-4.1270853847
C	4.9889838465	0.8143220808	-2.6140944320
C	5.6940464713	1.7806672117	-3.3836886936
H	6.0263729591	1.5060252271	-4.3803397136
C	5.9454311587	3.0358162930	-2.8849772452
H	6.4818911424	3.7650143161	-3.4821247831
C	5.4966934341	3.3831692540	-1.5876122087
H	5.6833846687	4.3802833992	-1.2043858620
C	4.8148121267	2.4707770431	-0.8177962959
H	4.4635251654	2.7543367177	0.1670097692
C	4.5416975526	1.1593033825	-1.3014603270
C	-2.2293234323	-1.6187274084	0.6811740618
C	-3.6154858912	-1.4037713116	0.4657932107
C	-4.5240440688	-2.3918284025	0.8618871733

H	-5.5799903972	-2.2193063106	0.6892801078
C	-4.0910786523	-3.5785305375	1.4408661819
H	-4.8103684456	-4.3312468192	1.7430523789
C	-2.7294934697	-3.8122394914	1.5919400528
H	-2.3758167458	-4.7548844372	1.9929643341
C	-1.7809173784	-2.8571946408	1.2082941612
C	-4.2127970636	-0.1911603521	-0.1732744840
C	-5.0658062485	0.5922649754	0.5722348951
H	-5.2357553355	0.3442523945	1.6151644167
C	-5.6999283480	1.7282642535	0.0139303510
H	-6.3491702879	2.3359344066	0.6350834937
C	-5.4894227775	2.0542677113	-1.3014053509
H	-5.9652825051	2.9256789009	-1.7402120830
C	-4.6494408881	1.2487836000	-2.1211307582
C	-4.4390104413	1.5603995648	-3.4923490351
H	-4.9254769129	2.4379414314	-3.9058508337
C	-3.6196012472	0.7849029388	-4.2754777392
H	-3.4519123668	1.0411069894	-5.3152027068
C	-2.9795566032	-0.3495749525	-3.7212815687
H	-2.3283028975	-0.9551200716	-4.3411718326
C	-3.1730110052	-0.6813636550	-2.4016819489
H	-2.6854049057	-1.5587341936	-1.9928661685
C	-4.0034638363	0.1080691916	-1.5601871878
C	-0.3530409904	-3.2742042053	1.3729293060
C	0.1456430875	-3.4616937732	2.6429106661
H	-0.4842599295	-3.2322312722	3.4949534438
C	1.4654914797	-3.9276847848	2.8532777108
H	1.8380583168	-4.0355299779	3.8659063343
C	2.2615520169	-4.2452840796	1.7824112180
H	3.2734206609	-4.6083532509	1.9319674961
C	1.7693920165	-4.1162143968	0.4536167701
C	2.5615237766	-4.4892194353	-0.6680493012
H	3.5598870888	-4.8780353770	-0.4927991762
C	2.0785092321	-4.3586005693	-1.9479637882
H	2.6888223136	-4.6514416434	-2.7953338144
C	0.7816132955	-3.8342710195	-2.1657114614
H	0.4154579027	-3.7115219732	-3.1782789058
C	-0.0102977236	-3.4777056402	-1.1012299717
H	-1.0069107176	-3.0910695339	-1.2798454521
C	0.4523667463	-3.6122480753	0.2363608541

Supplementary References

1. Kays (née Coombs), D. L., Cowley, A. R. Monomeric, two-coordinate Mn, Fe and Co(II) complexes featuring 2,6-(2,4,6-trimethylphenyl)phenyl ligands. *Chem. Commun*., 1053-1055 (2007).
2. Hino, S., Olmstead, M. M., Fettinger, J. C., Power, P. P. Synthesis and structure of two lithium terphenyls and a “halide rich” terphenyl lithium species. *J. Organomet. Chem.*, **690**, 1638-1644 (2005).
3. Rabe, G. W., Sommer, R. D., Rheingold, A. L. Synthesis and X-ray crystal structure determination of tetrahydrofuran adducts of two terphenyllithium compounds. *Organometallics*, **19**, 5537-5540 (2000).
4. Cosier, J., Glazer, A. M. A nitrogen-gas-stream cryostat for general X-ray diffraction studies. *J. Appl. Crystallogr.*, **19**, 105-107 (1986).
5. CrysAlisPRO Oxford Diffraction/Agilent Technologies UK Ltd Yarnton England.
6. Sheldrick, G. M. Crystal structure refinement with SHELXL. *Acta Crystallogr. Sect. C Struct. Chem.*, **71**, 3-8 (2015).
7. Sheldrick, G. M. A short history of SHELX. *Acta Crystallogr. Sect. A Found. Crystallogr.*, **64**, 112-122 (2008).
8. Sheldrick, G. M. SHELXT - Integrated space-group and crystal-structure determination. *Acta Crystallogr. Sect. A Found. Crystallogr.*, **71**, 3-8 (2015).
9. Dolomanov, O. V, Bourhis, L. J., Gildea, R. J., Howard, J. A. K., Puschmann, H. OLEX2: a complete structure solution, refinement and analysis program. *J. Appl. Cryst*, **42**, 339-341 (2009).
10. http://checkcif.iucr.org/
11. Gridley, B. M. *et al.* Low-coordinate cobalt(II) terphenyl complexes: precursors to sterically encumbered ketones. *Chem. Commun.*, **48**, 8910-8912 (2012).
12. Chai, J. –D., Head-Gordon, M. Long-range corrected hybrid density functionals with damped atom–atom dispersion corrections. *Phys. Chem. Chem. Phys*., **10**, 6615-6620 (2008).
13. Hariharan, P. C., Pople, J. A. The influence of polarization functions on molecular orbital hydrogenation energies. *Theoret. Chim. Acta,* **28**, 213-222 (1973).
14. Malmqvist, P. –Å., Rendell, A., Roos, B. The restricted active space self-consistent-field method, implemented with a split graph unitary group approach. *J. Phys. Chem.*, **94**, 5477-5482 (1990).

15. Y. Shao, *et al.* Advances in molecular quantum chemistry contained in the Q-Chem 4 program package. *Mol. Phys.,* **113**, 184-215 (2015).
16. Adamo, C. Barone, V. Toward reliable density functional methods without adjustable parameters: The PBE0 model. *J. Chem. Phys.,* **110**, 6158-6170 (1999).
17. V. Barone, in Recent Advances in Density Functional Methods, Part 1 (ed. D. P. Chong), World Scientific, Singapore, p. 287 (1995).
18. F. Neese, The ORCA program system. Wiley Interdisciplinary Reviews: Computational Molecular Science, **2**, 73-78 (2012).
19. Hay, P. J., Wadt, W. R. Ab initio effective core potentials for molecular calculations. Potentials for K to Au including the outermost core orbitals. *J. Chem. Phys.* **82**, 299-310 (1985).
20. Hehre, W. J., Ditchfield, R., Pople, J. A. Self-Consistent Molecular Orbital Methods. XII. Further Extensions of Gaussian-Type Basis Sets for Use in Molecular Orbital Studies of Organic Molecules. *J. Chem. Phys.,* **56**, 2257 (1972).
21. Becke, A. D. Density-functional thermochemistry. III. The role of exact exchange. *J. Chem. Phys.,* **98**, 5648-5652 (1993).
22. Dolg, M., Wedig, U., Stoll, H., and H. Preuss, Energy-adjusted *ab initio* pseudopotentials for the first row transition elements. *J. Chem. Phys.* **86**, 866-872 (1987).
23. Krishnan, R., Binkley, J. S., Seeger, R., Pople, J. A. Self-consistent molecular orbital methods. XX. A basis set for correlated wave functions. *J. Chem. Phys.,* ***72***, 650-654 (1980).
24. Truong, T. N., Stefanovich, E. V. A new method for incorporating solvent effect into the classical, ab initio molecular orbital and density functional theory frameworks for arbitrary shape cavity. *Chem. Phys. Lett.,* **240**, 253-260 (1995).
25. Barone, V., Cossi, M. Quantum calculation of molecular energies and energy gradients in solution by a conductor solvent model. *J. Phys. Chem. A,* **102**, 1995-2001 (1998).
26. Cossi, M., Rega, N., Scalmani, G., Barone, V. Energies, structures, and electronic properties of molecules in solution with the C-PCM solvation model. *J. Comput. Chem.,* **24**, 669-681 (2003).
